# Ethnomedicinal Uses, Geographical Distribution, Botanical Description, Phytochemistry, Pharmacology, and Quality Control of *Laportea bulbifera* (Sieb. et Zucc.) Wedd.: A Review

**DOI:** 10.3390/molecules29010042

**Published:** 2023-12-20

**Authors:** Jiaxin Feng, Guangqing Xia, Junyi Zhu, Li Li, Hao Zang

**Affiliations:** 1 Green Medicinal Chemistry Laboratory, School of Pharmacy and Medicine, Tonghua Normal University, Tonghua 134002, China; 13630304082@163.com (J.F.); qingguangx@thnu.edu.cn (G.X.); swx0527@163.com (J.Z.); 2 College of Pharmacy, Yanbian University, Yanji 133002, China; 3 Key Laboratory of Evaluation and Application of Changbai Mountain Biological Gerplasm Resources of Jilin Province, Tonghua 134002, China

**Keywords:** *Laportea bulbifera* (Sieb. et Zucc.) Wedd., chemical composition, geographical distribution, morphological description, quality control, pharmacological effects

## Abstract

*Laportea bulbifera* (Sieb. et Zucc.) Wedd. (*L. bulbifera*) is a significant plant in the *Laportea* genus. Traditionally, it has been used in ethnomedicine for treating various conditions such as rheumatic arthralgia, fractures, falling injuries, nephritis dropsy, limb numbness, pruritus, fatigue-induced internal imbalances, and irregular menstruation. Modern pharmacological studies have confirmed its therapeutic potential, including anti-inflammatory, immunosuppressive, analgesic, and anti-rheumatoid arthritis properties. To gather comprehensive information on *L. bulbifera*, a thorough literature search was conducted using databases like Web of Science, PubMed, ProQuest, and CNKI. This review aims to provide a comprehensive understanding of *L. bulbifera*, covering various aspects such as ethnomedicinal uses, geographical distribution, botanical description, phytochemistry, pharmacology, and quality control. The goal is to establish a solid foundation and propose new research avenues for exploring and developing potential applications of *L. bulbifera*. So far, a total of one hundred and eighty-nine compounds have been isolated and identified from *L. bulbifera*, including flavonoids, phenolics, nitrogen compounds, steroids, terpenoids, coumarins, phenylpropanoids, fatty acids and their derivatives, and other compounds. Notably, flavonoids and fatty acids have demonstrated remarkable antioxidant and anti-inflammatory properties. Additionally, these compounds show promising potential in activities such as analgesia, hypoglycemia, and hypolipidemia, as well as toxicity. Despite extensive fundamental studies on *L. bulbifera*, further research is still needed to enhance our understanding of its mechanism of action and improve quality control. This requires more comprehensive investigations to explore the specific material basis, uncover new mechanisms of action, and refine quality control methods related to *L. bulbifera*. By doing so, we could contribute to the further development and utilization of this plant.

## 1. Introduction

**Figure 1:**
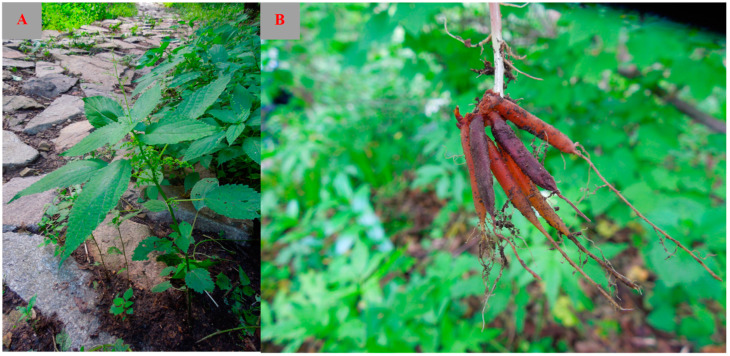
Morphology of *Laportea bulbifera*: aboveground part (**A**) and root (**B**).

*Laportea bulbifera* (Sieb. et Zucc.) Wedd. (*L. bulbifera*) (Figure 1) is an important plant in the *Laportea* genus. It is referred to by various names, including *Laportea elevata*, *Laportea terminalis*, and *Laportea sinensis*. Currently, a variety of active ingredients have been isolated from *L. bulbifera*, such as flavonoids [1,2,3], coumarins [1,4,5], phenolic acids [6], phenylpropanoids [7,8], steroids [1,9,10], aliphatic acids [5,8], nitrogen compounds [8,11], and other compounds. Modern pharmacological studies have demonstrated that extracts and monomeric compounds from *L. bulbifera* possess anti-inflammatory [12,13], immunosuppressive [14], analgesic [15], and anti-rheumatoid arthritis properties [16], with particular emphasis on its anti-inflammatory and anti-rheumatoid arthritis effects.

Among the ethnic medicines in Guizhou Province in southwest China that have been incorporated into the national drug standards, various preparations containing *L. bulbifera* have been developed. These include Runzao Antipruritic Capsules, Liuwei Shangfuning Ointments, Fufang Shangfuning Ointments, and Tongluo Guzhining Ointments. The cultivation and utilization of *L. bulbifera* have become crucial endeavors in Guizhou’s ethnic medicine pillar industry, possessing distinctive regional resource advantages and development potential [16]. Runzao Antipruritic Capsules, in particular, have gained significant popularity in the Chinese market due to their unique therapeutic effect in treating skin itching caused by blood deficiencies in the elderly [17]. This inclusion in the Report on the Scientific and Technological Competitiveness of Large Varieties of Traditional Chinese Medicine showcases their popularity [18]. Additionally, the young leaves of *L. bulbifera* are edible, and the stem fibers are durable and suitable for use in textile production [19].

Despite existing research that has summarized the phytochemistry and pharmacology of *L. bulbifera* [20], there are significant gaps in the coverage. These gaps include the incomplete classification of components, a partial listing of constituents, and the lack of information concerning the chemical structure, exact theoretical molecular weight, and characterization method for these components. Furthermore, the mechanisms underlying the pharmacological effects are often insufficiently detailed and clarified.

In contrast, our review addresses these deficiencies by reporting a total of one hundred and eighty-nine components and providing structural information for each compound, including the name, formula, exact theoretical molecular weight, characterization method, references, and source. Additionally, our review introduces a different classification of pharmacological research compared to the previous report. Importantly, we incorporate the latest research findings on *L. bulbifera*, resulting in an up-to-date and comprehensive perspective.

Therefore, the objective of our review is to bridge these gaps by providing a comprehensive assessment of the ethnomedicinal uses, geographical distribution, botanical description, phytochemistry, pharmacology, and quality control of *L. bulbifera*. This review aims to serve as a valuable reference for future investigations into *L. bulbifera*, as well as offering new insights into the rational utilization of *L. bulbifera* resources and the efficient development of related products.

## 2. Ethnomedicinal Uses

*L. Bulbifera*, also known as “reib ndad gunb” or “uab detdend” in the Miao language, is widely used as a traditional medicine by ethnic minorities in Guizhou Province, Hubei Province, and Guangxi Zhuang Autonomous Region, China. These communities include the Miao, Buyi, Tujia, Zhuang, and Yao. During the autumn season, the roots are harvested and then sun-dried after removing the stems, leaves, and soil. *L. Bulbifera* has a pungent flavor and a hot nature, making it suitable for treating conditions related to the cold meridian [21]. Its primary functions include clearing the blood network and nervous network [6]. For internal use, it is typically decocted with water at a dosage of 9–15 g. When using fresh products, the dosage should be doubled. Alternatively, it can be soaked in Chinese Baijiu. For external application, an appropriate amount can be used for washing or applied externally after being mashed. Its effects encompass dispelling wind and dampness, promoting blood circulation, and removing stasis. It is particularly effective in clearing the food channel, strengthening the spleen, and eliminating accumulated food. Common applications include the treatment of rheumatic arthralgia, fractures, falling injuries, nephritis dropsy, limb numbness, pruritus, fatigue-induced internal imbalances, and irregular menstruation. Additionally, Zhuang doctors often use it to address infantile malnutrition in children and urinary tract stones. The following are some specific prescriptions that involve *L. Bulbifera*: (1) To treat rheumatism and numbness, decoct 15 g of *L. Bulbifera* with water, take the water decoction orally, and use the water decoction to wash the affected area. (2) For rheumatic arthralgia, soak 15 g of *L. Bulbifera* and 9 g of *Acanthopanacis gracilistylus* in Chinese Baijiu before consuming. (3) For falling injuries, grind the dried roots into powder and take 6 g of Chinese Baijiu before bedtime. (4) To treat urticaria, decoct 6–9 g of *L. Bulbifera* with water and take the water decoction orally. For pediatric use, the dosage should be appropriately reduced. (5) To alleviate body deficiency and swelling, take 9–15 g of *L. Bulbifera* and 250 g of pork. Stew them together and consume the soup and meat once a day for 2–3 days. (6) For cough, decoct 20–30 g of *L. Bulbifera* with water and take the water decoction orally. (7) For anemofrigid cold and cough, decoct 30 g of *L. Bulbifera* with water and take the water decoction orally [21].

## 3. Geographical Distribution

**Figure 2:**
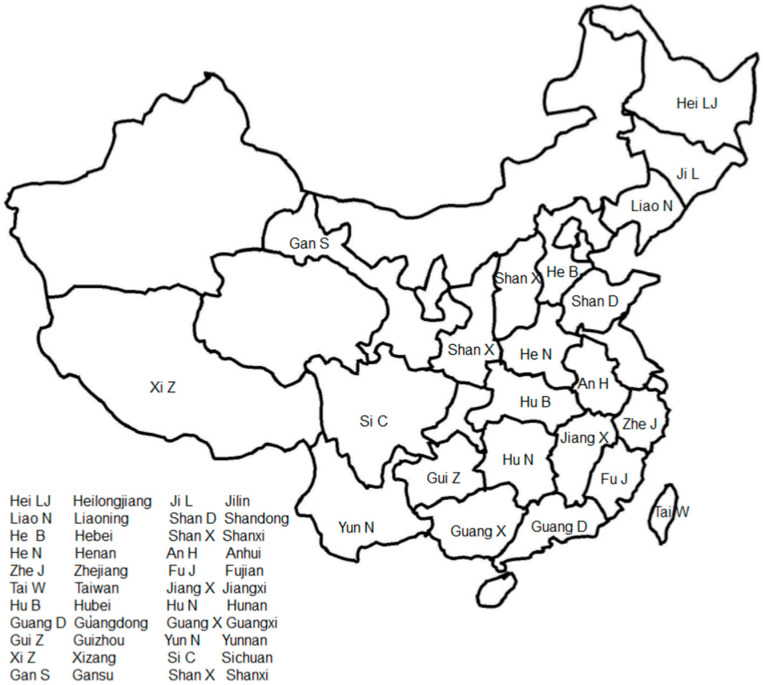
The general geographical distribution of *Laportea bulbifera* in China.

*L. bulbifera* is distributed across various regions in China, including Heilongjiang, Jilin, Liaoning, Shandong, Hebei, Shanxi, Henan, Anhui, Zhejiang, Fujian, Taiwan, Jiangxi, Hubei, Hunan, northern Guangdong, Guangxi, Guizhou, Yunnan, Xizang, Sichuan, Gansu, and Shaanxi. Figure 2 illustrates the general geographical distribution of *L. bulbifera* in China. It can also be found in Japan, North Korea, Russia, Sikkim, India, Sri Lanka, and Java Island in Indonesia. This plant grows in hillside forests and on semi-shady slopes at altitudes of 1000–2400 m [22].

## 4. Botanical Description

The female perianth has four segments, and the male perianth has 4–5 segments. The ovary has a pistil stalk, and the stigma is filiform, measuring 2–4 mm in length. Initially, the ovary is upright and later becomes oblique. The achenes are round and obovate or nearly semicircular, oblique, flat, and 2–3 mm long with purplish-brown spots. The pistil stalk is retroflex, and two persistent perianth segments extend to the middle of the fruit. The fruit stalk has membranous wings, and sometimes, the fruit inflorescence is branched and winged, spoon-shaped, with a concave top. The flowering period is from June to August, and the fruiting period is from August to December [22,23].

*L. bulbifera* is a perennial herb. The root of *L. bulbifera* is long and conical or slender, spindle-shaped, and twisted, with a length ranging from 6 to 20 cm and a diameter of 3–6 mm. The surface has a grayish-brown to reddish-brown color, with fine longitudinal wrinkles and slender fibrous roots or fibrous root scars. It has a hard texture and is not easily broken, with a fibrous cross-section and a light reddish-brown color [21]. The stem is 0.4–1.5 m tall, with short hairs and a few stinging hairs. The bulbils are almost spherical, with a diameter of 3–6 mm. The leaves are alternate, ovate, elliptical, or lanceolate, measuring 8–16 cm in length and 3–6 cm in width. The apex is acuminate, the base is broadly cuneate or circular, and the margin is densely toothed. The lower surface is sparsely covered with short hairs and stinging hairs. Cystoliths are punctate, with three basal veins and 4–6 pairs of lateral veins. The petiole is 1.5–6 cm long, and the stipules are oblong-lanceolate, measuring 0.5–1 cm in length and being two-lobed. The inflorescence is paniculate, and the plant is monoecious. The male inflorescence is located in the upper leaf axil of the stem and measures 3–10 cm in length, while the female inflorescence is located at or near the top leaf axil, measuring 10–25 cm in length with a peduncle of 5–12 cm. The female perianth has 4 segments, and the male perianth has 4–5 segments. The ovary has a pistil stalk, and the stigma is filiform, measuring 2–4 mm in length. Initially, the ovary is upright and later becomes oblique. The achenes are round, obovate, or nearly semicircular, oblique, flat, and 2–3 mm long with purplish-brown spots. The pistil stalk is retroflex, and two persistent perianth segments extend to the middle of the fruit. The fruit stalk has membranous wings, and sometimes, the fruit inflorescence is branched and winged, spoon-shaped, with a concave top. The flowering period is from June to August, and the fruiting period is from August to December [22,23].

## 5. Phytochemistry

Over the years, numerous active compounds have been isolated and identified from the aerial parts or roots of *L. bulbifera*, particularly in recent times. As the importance and utilization of this plant increase, research on its components has also expanded. According to reports, a total of one hundred and eighty-nine compounds have been isolated or identified from *L. bulbifera*. These compounds can be categorized into nine groups, including flavonoids, phenolics, nitrogen compounds, steroids, terpenoids, coumarins, phenylpropanoids, fatty acids and their derivatives, as well as other compounds. This remarkable abundance of bioactive ingredients in *L. bulbifera* highlights its potential as a source for drug development and clinical applications.

### 5.1. Flavonoids

**Figure 3:**
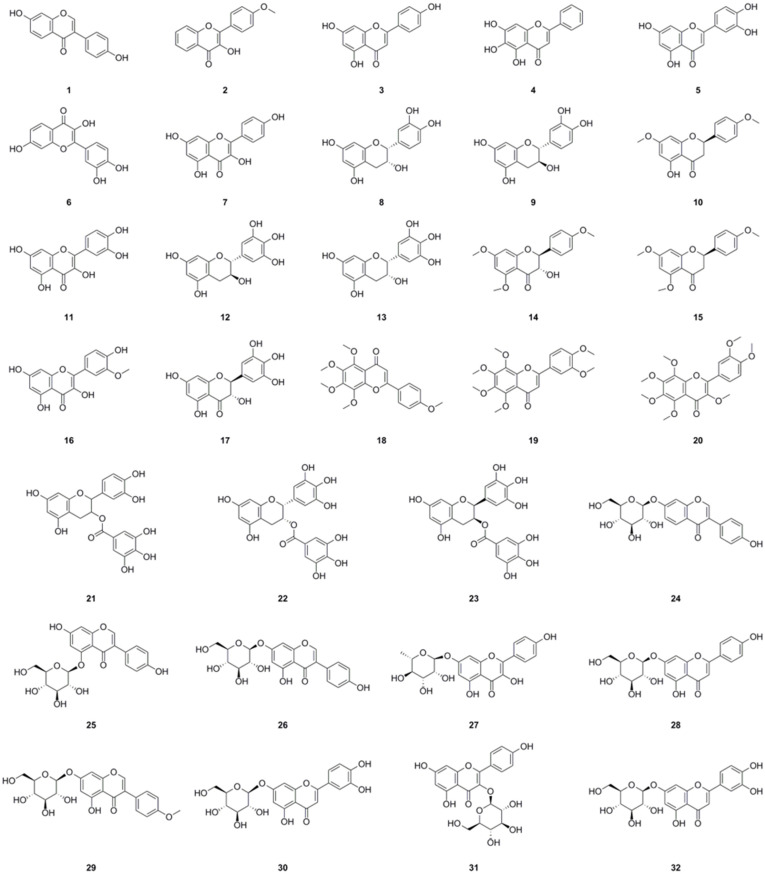

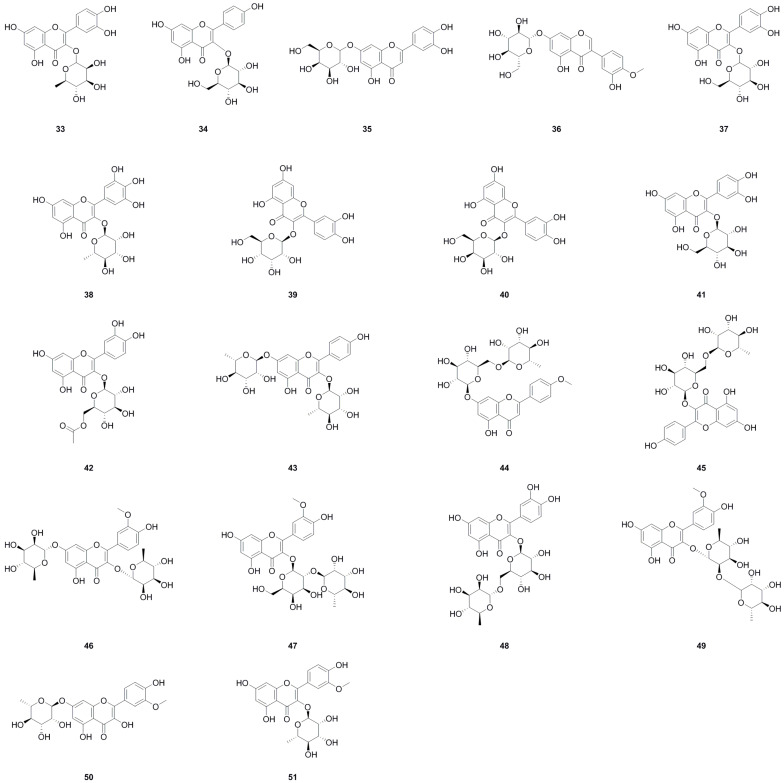
Chemical structures of flavonoids isolated from *Laportea bulbifera*. Chemical structures were drawn using ChemDraw Professional 15.0 software.

Among the compounds derived from *L. bulbifera*, flavonoids have received the most extensive research and were the earliest reported type. A total of fifty-one flavonoid components have been identified, consisting of twenty-three flavonoids and twenty-eight flavonoid glycosides (Table 1, Figure 3). The team from Dalian University isolated nine flavonoids and their glycosides from the aerial parts and the whole herb of *L. bulbifera*, respectively [2,24]. Additionally, five flavonoids were isolated from the aerial parts [11], while twenty-six flavonoids and their glycosides were obtained from the roots of *L. bulbifera* through bioassay-guided isolation [1]. Furthermore, HPLC-MS technology was used to identify seven flavonoids and their glycosides from the 95% ethanol extract of the roots [2]. Epigallocatechin was isolated from the whole herb [25], and rutin was isolated from the aerial parts [26]. Two flavonoid glycosides were also obtained from the whole herb [10], and four flavonoids and their glycosides were identified through UHPLC-ESI-Q-TOF-MS technology from the 70% ethanol extract [7]. Similarly, We employed the same technique to identify two flavonoid glycosides from the methanol extract of the roots [27].

Flavonoids are natural polyphenolic substances and secondary metabolites of plants. They possess remarkable antioxidant activity, which has been extensively investigated. This antioxidant activity aids in the prevention of damage caused by free radicals through scavenging reactive oxygen species (ROS), activating antioxidant enzymes, and inhibiting oxidases. Moreover, flavonoids elevate uric acid levels and exhibit metal-chelating activity to alleviate oxidative stress [28]. Studies have also indicated that flavonoids activate antioxidant pathways, thereby contributing to their anti-inflammatory effects. They inhibit the secretion of enzymes such as lysozymes and *β*-glucuronidase, as well as the secretion of arachidonic acid, thus reducing inflammatory reactions. Flavonoids such as apigenin (**3**), kaempferol (**7**), and (−)-epigallocatechin 3-*O*-gallate (**22**) play a role in modulating the expression and activation of various cytokines, including interleukin-1beta (IL-1*β*), tumor necrosis factor-alpha (TNF-α), interleukin-6 (IL-6), and interleukin-8 (IL-8). They also regulate the gene expression of several pro-inflammatory molecules, such as nuclear factor-kappaB (NF-*κ*B), activator protein-1 (AP-1), and intercellular adhesion molecule-1 (ICAM). Additionally, they inhibit pro-inflammatory enzymes such as inducible nitric oxide (NO) synthase, cyclooxygenase-2, and lipoxygenase [29].

### 5.2. Phenolics

**Figure 4:**
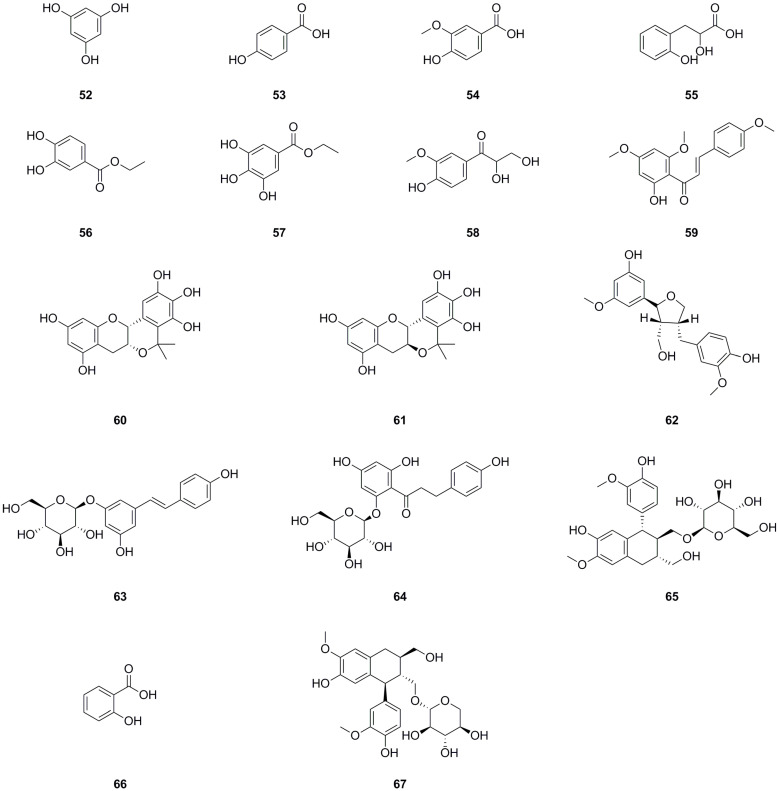
Chemical structures of phenolics isolated from *Laportea bulbifera.* Chemical structures were drawn using ChemdDaw Professional 15.0 software.

A total of sixteen phenolics have been isolated and identified from different parts of *L. bulbifera*, including the roots, aerial parts, and the whole herb (Table 2, Figure 4). Nine phenolics were obtained from the roots using bioassay-guided isolation [1]. Phloroglucinol (**52**) was isolated from the whole herb [25], C-veratroylglycol (**58**) was isolated from the roots [8], and vanillic acid (**54**) was also isolated from the roots [30]. Another study identified phenolics such as ethyl 3,4-dihydroxybenzoate (**56**), ethyl gallate (**57**), and (+)-isolariciresinol 9′-*O*-glucoside (**65**). Two phenolics, salicylic acid (**66**) and schizandriside (**67**), were identified from the methanol extract of the roots using UHPLC-ESI-Q-TOF-MS technology. Phenolics have demonstrated potent antioxidant, anti-inflammatory, and immunomodulatory activities [31], as well as hypolipidemic, hypoglycemic, and antihypertensive properties [32].

### 5.3. Nitrogen Compounds

**Figure 5:**
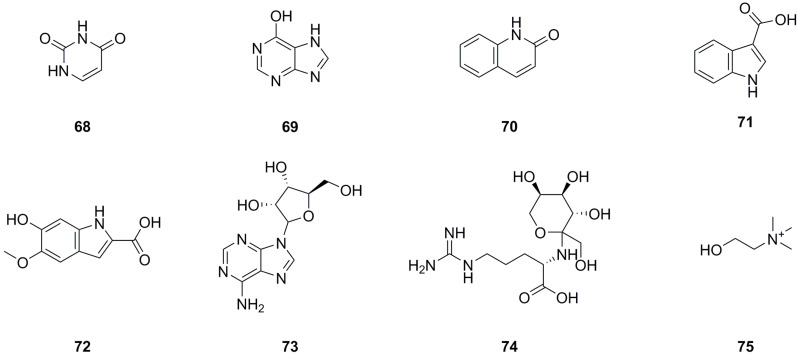
Chemical structures of nitrogen compounds isolated from *Laportea bulbifera.* Chemical structures were drawn using ChemDraw Professional 15.0 software.

Currently, eight nitrogen compounds have been isolated and identified from various parts of *L. bulbifera*, including the roots, aerial parts, and the whole herb (Table 3, Figure 5). Uracil (**68**), 6-hydroxypurine (**69**), 1H-indole-3-carboxylic acid (**71**), and 9-ribofuranosyladenine (**73**) were isolated from the aerial parts [11]. Quinolin-2(1H)-one (**70**) was identified from the aerial parts [5], and 6-hydroxy-5-methoxy-1H-indole-2-carboxylic acid (**72**) was identified from the 70% ethanol extract of the roots. N2-Fructopyranosylarginine (**74**) and choline were identified from the methanol extract of the roots using UHPLC-ESI-Q-TOF-MS [27].

### 5.4. Steroids

**Figure 6:**
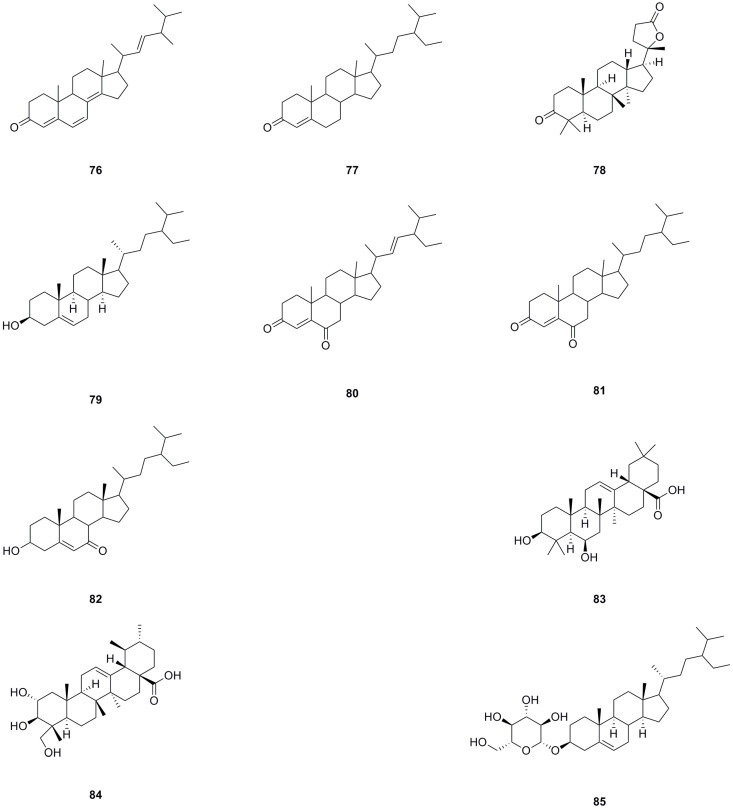
Chemical structures of steroids isolated from *Laportea bulbifera.* Chemical structures were drawn using ChemDraw Professional 15.0 software.

A total of ten steroids have been isolated and identified from different parts of *L. bulbifera*, including the roots, aerial parts, and the whole herb (Table 4, Figure 6). Ergosta-4,6,8(**14**),22-tetraen-3-one (**76**), sitostenone (**77**), stigmasta-4,22-diene-3,6-dione (**80**), and stigmast-4-ene-3,6-dione (**81**) were isolated from the whole herb [10]. (+)-Cabralealactone (**78**) and 7-keto-*β*-sitosterol (**82**) were isolated from the roots [1], while *β*-sitosterol (**79**) and *β*-daucosterol (**85**) were isolated from both the roots and aerial parts [9]. Sumaresinolic acid (**83**) and asiatic acid (**84**) were identified from the 70% ethanol extract of the roots through HPLC-MS analysis [8]. Among these steroids, compound **79** is the major compound and displays various biological activities, including immunomodulatory, anti-inflammatory, lipid-lowering, hepatoprotective, antioxidant, and anti-diabetic effects [33].

### 5.5. Terpenoids

**Figure 7:**
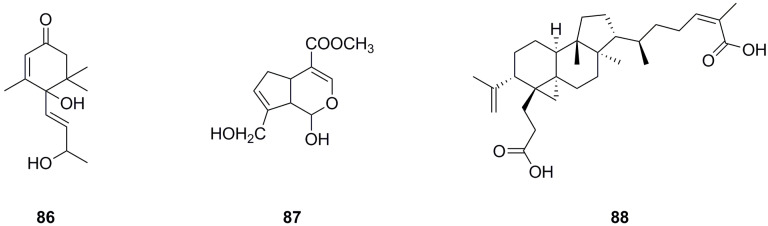
Chemical structures of terpenoids isolated from *Laportea bulbifera.* Chemical structures were drawn using ChemDraw Professional 15.0 software.

Only three terpenoids have been isolated and identified from the roots and aerial parts of *L. bulbifera* so far (Table 5, Figure 7). α-Ionol (**86**) was isolated from the aerial parts [11], while genipin (**87**) and nigranoic acid (**88**) were identified from the roots.

### 5.6. Coumarins

**Figure 8:**
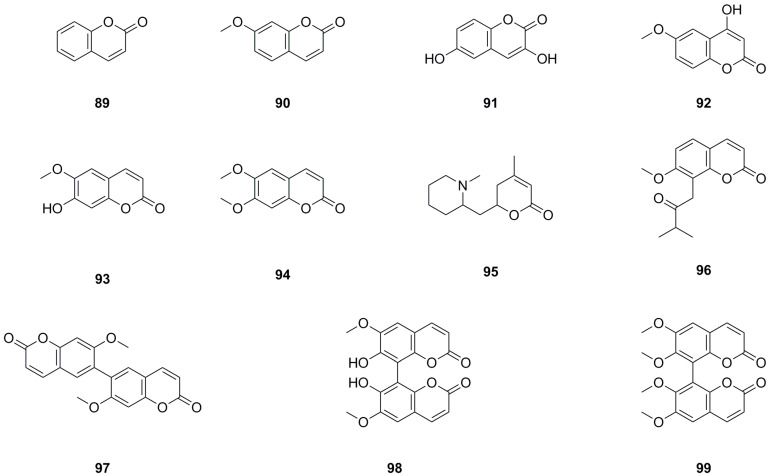
Chemical structures of coumarins isolated from *Laportea bulbifera.* Chemical structures were drawn using ChemDraw Professional 15.0 software.

Eleven coumarins have been isolated and identified from different parts of *L. bulbifera*, including the roots, aerial parts, and the whole herb (Table 6, Figure 8). The main categories of coumarins are simple coumarins and coumarin dimers. 7-Methoxy-2H-chromen-2-one (**90**) and scoparone (**94**) were isolated from the roots [1]. Five coumarins, including coumarin, were identified from the 70% ethanol extract of the whole herb using UHPLC-QTOF-MS/MS [5]. Scoparone (**94**) and three dimers, 7,7′-dimethoxy-6,6′-*bis*coumarin (**97**), 7,7′-dihydroxy-6,6′-dimethoxy-8,8′-*bis*coumarin (**98**), and 6,6′,7,7′-tetramethoxyl-8,8′-*bis*coumarin (**99**), were isolated from the roots [4]. Scopoletin (**93**) was isolated from both the aerial parts and the whole herb [6,11], while isomeranzin (**96**) was isolated from the whole herb [30]. Scopoletin (**93**) has antioxidant, anti-inflammatory, and neuroprotective properties [34]. Scoparone (**94**) possesses anti-inflammatory, antioxidant, anti-fibrotic, and hypolipidemic properties [35].

### 5.7. Phenylpropanoids

**Figure 9:**
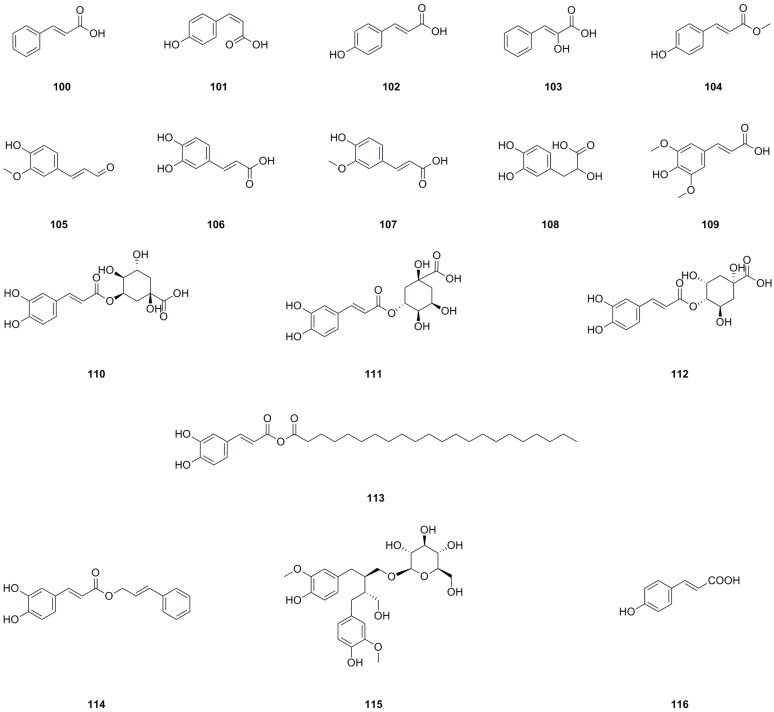
Chemical structures of phenylpropanoids isolated from *Laportea bulbifera.* Chemical structures were drawn using ChemDraw Professional 15.0 software.

Seventeen phenylpropanoids have been isolated and identified from the roots, aerial parts, or the whole herb of *L. bulbifera* (Table 7, Figure 9). Seven phenylpropanoids have been isolated and identified from the roots [8]. *trans*-*p*-Hydroxycinnamic acid (**102**), *cis*-hydroxycinnamic acid (**103**), and methyl-*trans*-4-hydroxycinnamate (**104**) have been isolated from the aerial parts [11].

Neochlorogenic acid (**110**), chlorogenic acid (**111**), and 4-*O*-caffeoylquinic acid have been identified from the 70% ethanol extract of the roots and the whole herb [7,26]. Caffeic acid cinnamyl ester (**114**), secoisolariciresinol 9-*O*-*β*-*D*-glucopyranoside (**115**), and (*E*)-4-coumaric acid (**116**) have been identified from the methanol extract of the roots by us [27]. Caffeic acid (**106**) has also been isolated from the roots [30]. Chlorogenic acid (**111**) is a significant compound with antioxidant, hepatoprotective, cardioprotective, anti-inflammatory, and free radical scavenging activities. Moreover, it has been found to modulate lipid metabolism and glucose levels [36]. Caffeic acid (**106**) is another important compound known for its antioxidant, immunomodulatory, and anti-inflammatory activities [37]. Danshensu (**108**) exhibits effects such as antioxidant properties, inflammation regulation, and lipidemia control [38].

### 5.8. Fatty Acids and Their Derivatives

**Figure 10:**
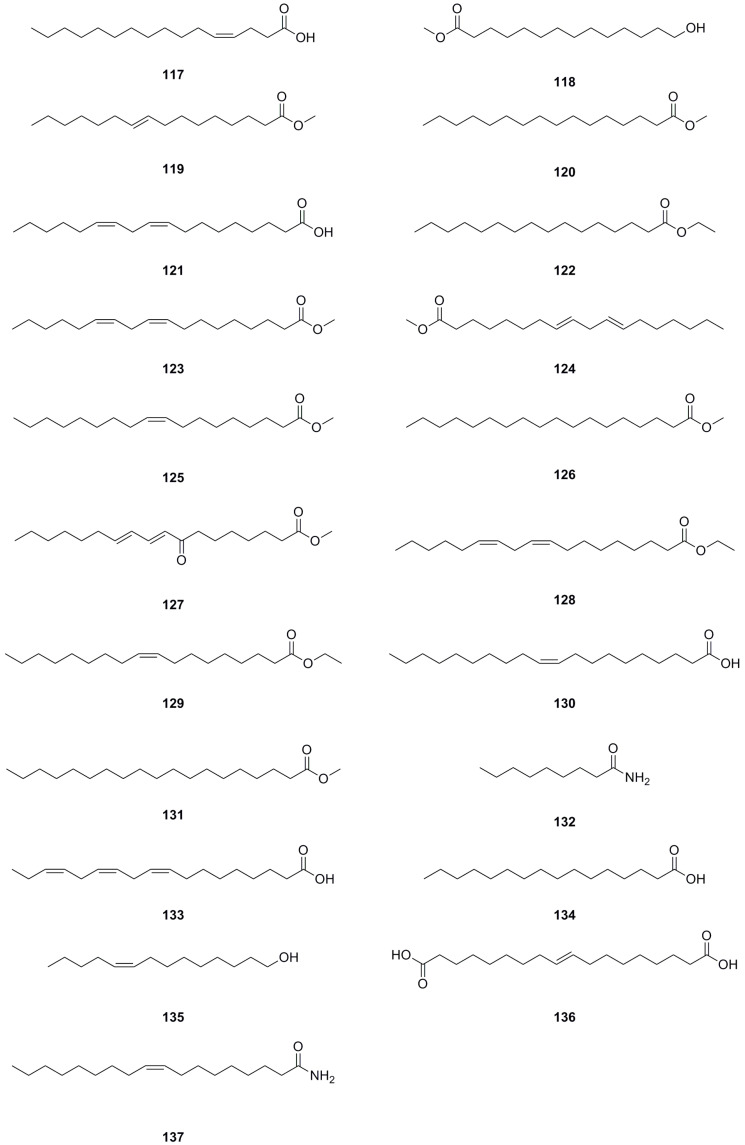

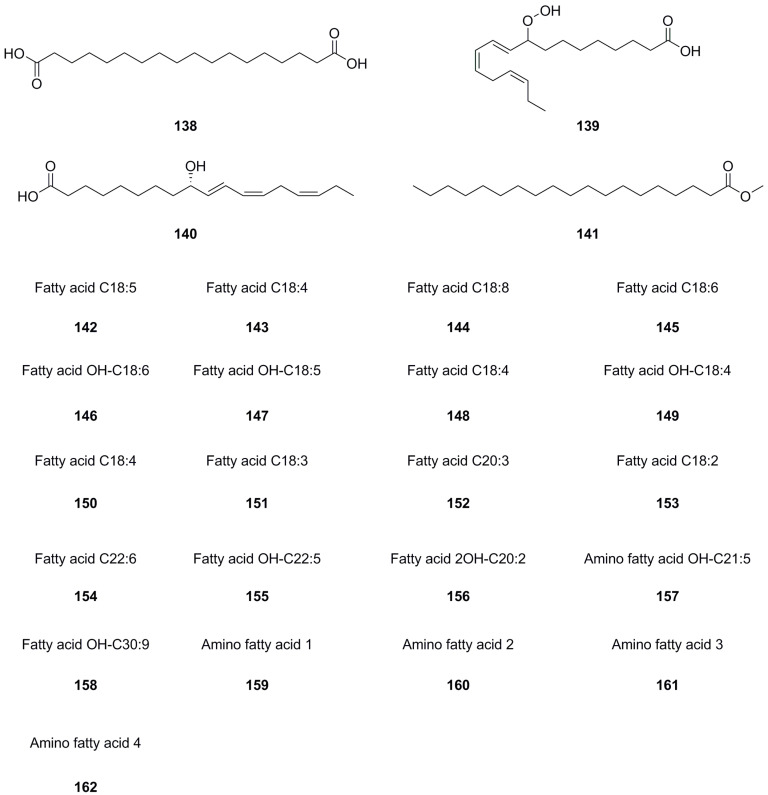
Chemical structures of fatty acids and their derivatives isolated from *Laportea bulbifera.* Chemical structures were drawn using ChemDraw Professional 15.0 software.

A total of forty-five fatty acids and their derivatives were isolated and identified from various parts of *L. bulbifera*, including the roots, aerial parts, and the whole herb (Table 8, Figure 10). These include saturated and unsaturated fatty acids, hydroxy fatty acids, amino fatty acids, fatty esters, and fatty amides. Fatty acids have shown potential in treating metabolic diseases such as type II diabetes, inflammatory diseases, and cancer [39,40]. Intake of linoleic acid (**121**) has been found to improve hyperlipidemia and reduce the incidence of type II diabetes [41]. Linolenic acid (**133**) possesses anti-metabolic syndrome, anticancer, anti-inflammatory, and antioxidant properties [42].

### 5.9. Others

**Figure 11:**
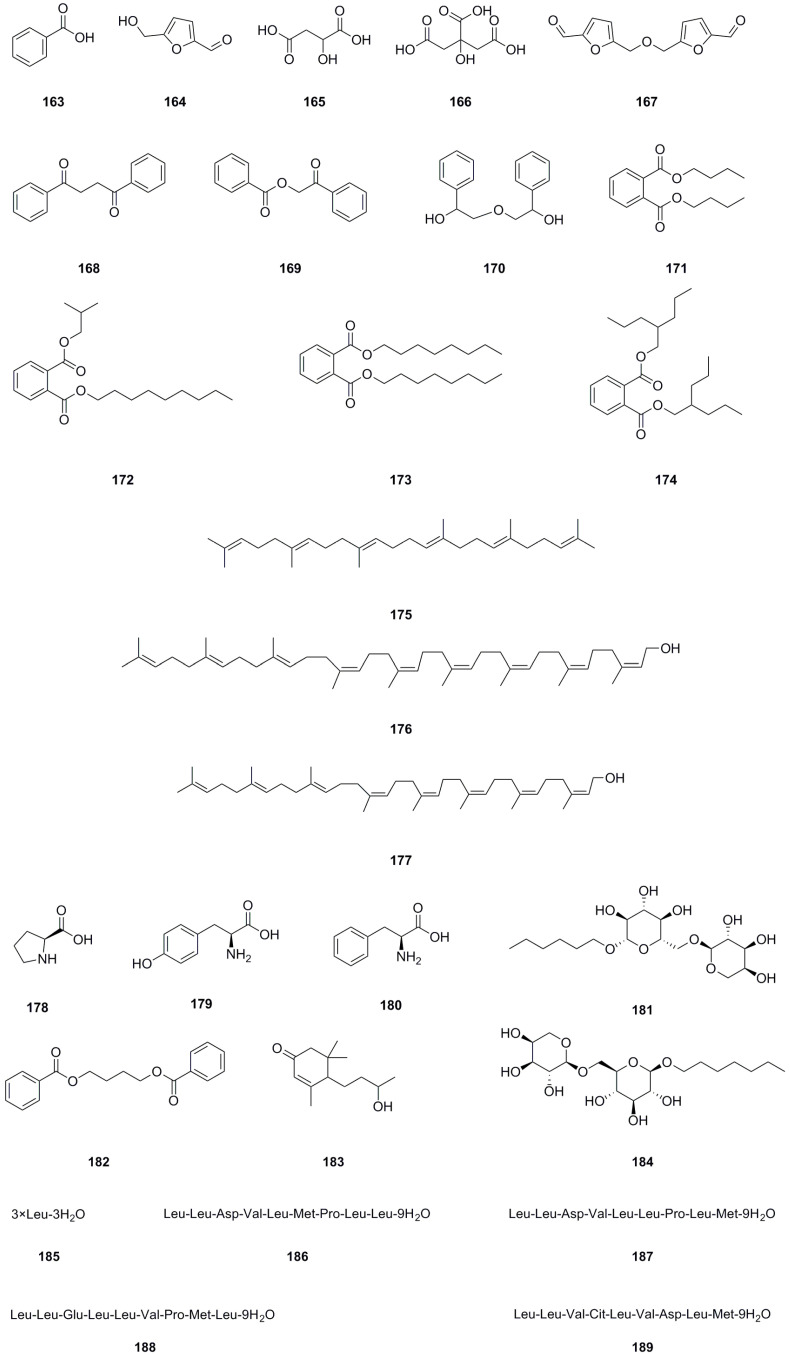
Chemical structures of others isolated from *Laportea bulbifera.* Chemical structures were drawn using ChemDraw Professional 15.0 software.

In addition to the previously mentioned compound types, twenty-seven other compound types have been isolated and identified from different parts of *L. bulbifera*, including the roots, aerial parts, and the whole herb (Table 9, Figure 11). The roots contain three organic acids: benzoic acid (**163**), malic acid (**165**), and citric acid (**166**) [8]. The whole herb contains four phthalate esters: dibutyl phthalate (**171**), phthalic acid, isobutyl nonyl ester (**172**), dioctyl phthalate (**173**), and *bis*(2-propylpentyl) phthalate (**174**) [25,30]. Squalene (**175**) has been isolated from the roots [1]. Betulaprenol 9 (**176**) and betulaprenol 8 (**177**) have been isolated from the whole herb [10]. The roots also contain three amino acids, *L*-proline (**178**), *L*-tyrosine (**179**), and phenylalanine (**180**), as well as two alkyl glycosides: creoside IV (**181**) and heptyl 6-*O*-*α*-*L*-arabinopyranosyl-*β*-*D*-glucopyranoside (**184**) [8]. Additionally, five oligopeptides (**185**–**189**) have been identified from the whole herb.

## 6. Quality Control

For a long time, *L. bulbifera* has mainly relied on wild resources. However, with the increasing popularity of traditional Chinese medicine based on it, the demand has been growing year by year. Simultaneously, the wild resources have been gradually depleted, and their quality is inconsistent, thus failing to meet the application needs. Therefore, it is crucial to conduct prompt research on quality control. It is worth mentioning that the “Quality Standards for Traditional Chinese Medicine and Ethnomedicine in Guizhou Province” includes documentation on the whole herb of *L. bulbifera*. This standard only provides information on its name, source, characteristics, identification, nature and flavor, channel tropism, main functions, usage, dosage, and storage. Among these, microscopic identification and thin-layer chromatography (TLC) are used for identification, with *β*-sitosterol serving as the reference substance [43]. Nevertheless, the level of quality control is relatively low because *β*-sitosterol is not a characteristic compound and cannot represent the medicinal material’s quality.

Pharmacognostic research on *L. bulbifera* has been conducted in various studies [44,45]. These studies involve morphological identification; microscopic identification of roots, stems, and leaves; and the use of isorhamnetin-3-*O*-*α*-*L*-rhamnopyranosyl-(1-2)-*β*-galactopyranoside (**47**) as a characteristic compound. Furthermore, a characteristic fingerprint of *L. bulbifera* was established using HPLC to effectively differentiate it from similar varieties [45]. Researchers have also developed TLC and HPLC methods utilizing rutin (**48**) as the characteristic component. By determining the rutin content in *L. bulbifera* from different regions, they are able to evaluate the medicinal material’s quality [26]. Additionally, a study has established an HPLC method for the determination of multiple indicators: epicatechin (**8**), catechin (**9**), (−)-gallocatechin (**12**), and epigallocatechin (**13**) in *L. bulbifera*. This simple method could be employed for the quality control of *L. bulbifera* [25]. Furthermore, there are reports on the simultaneous determination of eleven components (flavonoids and phenylpropanoids) in *L. bulbifera* using UHPLC-ESI-MS, which could be utilized for quality control [46]. This method is currently the most comprehensive for quality control purposes. Researchers have also examined the content of total active ingredients in *L. bulbifera*, such as total flavonoids [26], total polysaccharides [47], or total coumarins, to evaluate the medicinal material’s quality [48]. Moreover, scholars have investigated quality-related parameters, including water content, total ash content, acid-insoluble ash content, ethanol-soluble extractives, heavy metals, harmful elements, and organochlorine pesticide residues in *L. bulbifera* [25,26].

Research has shown that *L. bulbifera* is rich in coumarins and exhibits significant therapeutic effects on arthritis [16]. Moreover, studies indicate a high content of catechins in *L. bulbifera*, resulting in notable anti-inflammatory effects [1]. Additionally, research findings demonstrate that *L. bulbifera* has a high flavonoid content and diverse flavonoid types, displaying potent antioxidant activity [11]. Nevertheless, there is significant variation in the results of these studies on active ingredients, with minimal intersections. The underlying reason for this outcome remains unclear and may be attributed to differences in the origin, medicinal parts, and processing methods of *L. bulbifera*. Future research should focus on strengthening the investigation of its chemical components to elucidate the compounds responsible for its pharmacological effects. Consequently, a correlation model based on spectral efficacy was established to identify quality markers that better reflect the quality of *L. bulbifera*.

## 7. Pharmacological Effects

As a medicinal plant, modern pharmacological studies have demonstrated various pharmacological effects of *L. bulbifera*, including antioxidant, anti-inflammatory, analgesic, hypoglycemic, and hypolipidemic activities, as well as toxicity.

### 7.1. Antioxidant Activity

Both the water and ethyl acetate extracts (100 μg/mL) of the roots from *L. bulbifera*, along with the forty-six isolated compounds (10 μM) from the root, were subjected to a 2,2-diphenyl-1-picrylhydrazyl (DPPH) assay, and most of them exhibited good antioxidant activity [1]. The petroleum ether extract, ethyl acetate extract, and water extract (1 g/mL) from forty-three batches of *L. bulbifera* demonstrated excellent antioxidant activity [13]. A study utilized the DPPH assay to determine the average scavenging rate of different polar extracts (1 mg/mL). The results indicated that the ethyl acetate extract (87.6%) > water extract (63.3%) > petroleum ether extract (36.8%). The ethyl acetate extract was identified as the active antioxidant extract of *L. bulbifera* using SPSS software (Version 16.0) for variance analysis [26]. Yang et al. isolated five flavonoids, with isorhamnetin-3-*O*-*α*-*L*-rhamnoside (**51**), isorhamnetin-3,7-*O*-*α*-*L*-dirhamnoside (**46**), and isorhamnetin-3-*O*-*α*-rhamnosyl-(1-2)-rhamnoside (**49**) showing DPPH scavenging ability (EC_50_ value) at 45, 20, and 55 μg/mL, respectively, which are comparable to *L*-ascorbic acid (11 μg/mL) [3]. Our previous research demonstrated that the antioxidant capacity of *L. bulbifera* root is significantly stronger than that of the aerial part. Through twelve antioxidant experiments, the methanol extract of *L. bulbifera* root exhibited the best performance among the tested extracts. Additionally, this extract could serve as an oxidative stabilizer for olive oil and sunflower oil, and it also has a protective effect on oxidative imbalance-related liver damage in rats [26].

### 7.2. Anti-Inflammatory and Analgesic Effects

Inflammation is a common pathological process in clinical practice. It is a defensive response that the body generates after tissue damage or invasion by pathogenic factors. It is essential for the occurrence and development of many diseases. Therefore, research on anti-inflammatory drugs is highly significant [49]. The ethyl acetate extracts (100 μg/mL) derived from the roots of *L. bulbifera* demonstrated significant inhibitory activity against cyclooxygenase-2 (COX-2), with an inhibitory rate of 60.7%. Out of the forty-six compounds (10 μM) isolated from the ethyl acetate extract, twenty-three compounds exhibited inhibitory rates higher than 50%. Among these, thirteen compounds displayed strong inhibitory activity with IC_50_ values lower than 1 μM. Notably, compounds such as (−)-epicatechin-3-*O*-gallate (**21**), hyperoside (**40**), rutin (**48**), quercetin (**11**), fisetin (**6**), and luteolin (**5**) (with IC_50_ values ranging from 0.13 to 0.24 μM) showed optimal COX-2 inhibitory potency. The inhibitory activity of flavonoids against COX-2 is influenced by the number and position of phenolic hydroxyl groups [1].

In a study using lipopolysaccharide (LPS) to stimulate a mouse macrophage RAW264.7 model, the effects of different extracts from *L. bulbifera* roots on NO release and their anti-inflammatory activity were examined. Results revealed that the dichloromethane extract, ethyl acetate extract, and *n*-butanol extract at concentrations of 15.5, 31.25, and 62.5 μg/mL, respectively, exerted a significant impact on NO release, with statistically significant differences observed. At a concentration of 62.5 μg/mL, the inhibitory effects of the petroleum ether extract, dichloromethane extract, ethyl acetate extract, and *n*-butanol extract on NO release were 11.42%, 21.01%, 33%, and 26.96%, respectively. Specifically, the ethyl acetate extract exhibited the most pronounced effect on NO release, and its impact was dose-dependent, demonstrating excellent anti-inflammatory activity [30]. Inflammatory cell models (RAW264.7) were utilized to evaluate the anti-inflammatory activities. Additionally, the petroleum ether extract (0.2, 2, 20 μg/mL) from *L. bulbifera* was assessed for its TNF-*α* inhibition activity. Further analysis is warranted for the thirty-five batches of petroleum ether extract exhibiting therapeutic effects under 2 μg/mL [13]. Several reports have explored the use of total coumarins derived from *L. bulbifera* roots (20, 40, and 60 mg/kg) to treat type II collagen-induced arthritis in Balb/c mice. The results demonstrated that treatment with total coumarins (60 mg/kg) led to a significant and dose-dependent reduction in clinical arthritis score and paw swelling. Pathological changes indicated that total coumarins protected tissues against bone destruction. This protective effect was associated with a considerable decrease in the production of IFN-*γ* and IL-2, an increase in IL-10 and TGF-*β*, and the suppressive expression of T-bet in dendritic cells. Additionally, total coumarins induced the generation of CD4^+^ CD25^+^ Treg cells expressing the Foxp3 phenotype. The dendritic cells treated with total coumarins displayed low expression of MHC class II and CD86 molecules, as well as reduced levels of IL-12p70. In summary, total coumarins exhibit significant protective effects and warrant further investigation and development as a potential anti-arthritis drug [16].

To evaluate the anti-rheumatoid arthritis effects of the serum, the human rheumatoid arthritis fibroblast-like synoviocyte line MH7A was cultured and treated with TNF-*α* (50 ng/mL) in vitro. The serum containing the whole herb of *L. bulbifera* was used to determine the proliferation and levels of inflammatory cytokines, such as prostaglandin E2 (PGE2), IL-1*β*, and IL-6, in the MH7A cells. The active components were identified based on the peak areas of common peaks and the results of the anti-rheumatoid arthritis effect test. The serum containing *L. bulbifera* significantly inhibited the proliferation of TNF-*α*-activated MH7A cells and the expression of PGE2, IL-6, and IL-1*β.* Thirty newly generated compounds were detected in the drug-containing serum. Among them, eight components were determined to enter the bloodstream as prototypes, and twelve components showed significant correlation with the pharmaceutical effect. Neochlorogenic acid (**110**), cryptochlorogenic acid (**112**), and chlorogenic acid (**111**) made significant contributions to the anti-rheumatoid arthritis activity [50].

The results of the experiment on anti-inflammatory activity showed that the swelling inhibition rate in mice treated with the 70% ethanol extract (20 g raw medicine/kg) of the whole herb from *L. bulbifera* was comparable to that of the positive group, with an inhibition rate greater than 50%. This inhibitory effect was better than that of the water extract. The test on analgesic activity showed that both the 70% ethanol extract group (20 g raw medicine/kg) and the water extract group (20 g raw medicine/kg) from the whole herb of *L. bulbifera* had an inhibitory effect on the number of twisting times in mice, but the former had a better effect. The experimental results also demonstrated that the pain threshold of mice increased by 34.2% after administration of the 70% ethanol extract (20 g raw medicine/kg), indicating its superior central analgesic effect caused by thermal stimulation compared to that of the water extract [25]. Studies also revealed that the ethyl acetate extract of *L. bulbifera* obtained similar results. It was found that the ethyl acetate extract could dose-dependently inhibit the proliferation of splenic T lymphocytes and the secretion of IL-2 and IFN-*γ* in the cell culture supernatant. These findings indicate that the ethyl acetate extract has a certain immunosuppressive effect and serves as the material basis for *L. bulbifera*’s anti-rheumatoid arthritis effect [15].

A study investigated the differences in intestinal absorption characteristics of *L. bulbifera* extract between normal and rheumatoid arthritis pathological states in rats. The absorption concentration of *L. bulbifera* extract was 5.0 mg/mL, and the UHPLC-MS/MS technique was used to detect the content of eight indicator components in the extract. The results revealed that all eight indicator components in the extract could be absorbed into the intestinal sac in a linear manner. The cumulative absorption time curve for each component showed a progressive increase without reaching saturation, suggesting a zero-order absorption rate process. It is suggested that the possible absorption mode for each component is passive diffusion, which provides a theoretical foundation for the development of oral dosage forms. Under normal conditions, the ileum (except for chlorogenic acid) exhibited the highest absorption of various components, while under pathological conditions, the duodenum showed the highest absorption. Additionally, the overall absorption of the eight components in each intestinal segment of rats with rheumatoid arthritis was higher than that of normal rats, suggesting that rheumatoid arthritis may alter the specific site of drug absorption [51].

In another study, the inhibitory effect of four isolated steroids from the whole herb of *L. bulbifera* on NO activity was evaluated using a mouse RAW264.7 cell model. The results indicated that the four steroid compounds (50 μg/mL) significantly reduced the production of NO in the model cells, with inhibition rates ranging from 27.41% to 40.10%. Among them, ergosterone exhibited the highest efficacy, suggesting that steroids may contribute to the anti-inflammatory properties of *L. bulbifera* [10].

A study utilized the LPS assay to determine the average anti-inflammatory activity of different polar extracts (1 mg/mL). The results showed that the petroleum ether extract (15.38%) exhibited the highest anti-inflammatory activity, followed by the ethyl acetate extract (7.91%) and the water extract (2.60%). The petroleum ether extract was identified as the active anti-inflammatory extract of *L. bulbifera* using SPSS software (Version 16.0) for variance analysis [26]. Another report also confirmed the potent anti-inflammatory effects of the petroleum ether extract [13]. Research findings suggest that (*E*)-4-coumaric acid (**116**) and caffeic acid (**106**) in *L. bulbifera* possess anti-inflammatory activity and can be absorbed into the bloodstream. These components are likely to be the effective anti-inflammatory compounds of *L. bulbifera* [8].

The results of a different study demonstrated that the ethyl acetate extract from *L. bulbifera* (at concentrations of 0.5, 1.0, and 1.5 mg/10 g) effectively inhibited the onset of inflammation and joint tissue lesions. It exhibited a favorable therapeutic effect on rheumatoid arthritis, as evidenced by the arthritis index, arthritis incidence rate, spleen index, toe swelling, and pathological photos. The ethyl acetate extract (at concentrations of 0.5, 1.0, and 1.5 mg/10 g) did not influence changes in surface antigens of dendritic cells, but it reduced the expression of T-bet and inhibited IFN-*γ* secretion while promoting IL-10 secretion. It also affected T cells by inhibiting T-bet expression and promoting GATA-3 expression, thereby enhancing the secretion of IL-4 and IL-10 while inhibiting the expression of IFN-*γ* and IL-2 to prevent the onset of rheumatoid arthritis [52].

In a study investigating the effects of total coumarins from *L. bulbifera* on mice with dextran sulfate sodium-induced colitis, it was found that intervention with different doses of total coumarins (37.5, 75, 150 mg/kg) significantly improved colitis symptoms. This improvement was characterized by stable weight gain, reduced damage to the intestinal mucosa, decreased infiltration of inflammatory cells, and the absence of diarrhea or bloody stools. Further research revealed that total coumarins were able to regulate the expression of pro-inflammatory and anti-inflammatory cytokines, as well as reduce the levels of TLR4 and NF-*κ*B in colon tissue. Moreover, no common adverse reactions such as weight loss, infection, or organ damage were observed during the administration of total coumarins. Therefore, this study provides a theoretical foundation for the development and usage of total coumarins of *L. bulbifera* as immunosuppressants [12].

The immunosuppressive activity of various compounds was assessed using the Cell Counting Kit-8 assay, and the results showed that 6,6′,7,7′-tetramethoxyl-8,8′-*bis*coumarin (**99**), 7,7′-dihydroxy-6,6′-dimethoxy-8,8′-*bis*coumarin (**98**), 7,7′-dimethoxy-6,6′-*bis*coumarin (**97**), and scoparone (**94**) exhibited immunosuppressive activity, with compound **99** showing particularly strong effects. Additionally, compound **99** (IC_50_, 5.19 × 10^−4^ mol/L) significantly enhanced the differentiation of CD4^+^CD25^+^Foxp3^+^ T regulatory cells compared to the normal control, as evidenced by FACS analysis. Therefore, compound **99** possesses specific immunosuppressive properties and holds potential as a therapeutic strategy for autoimmune diseases [4].

In another study, the immunosuppressive effects of the ethyl acetate extract from *L. bulbifera* were investigated in a murine model of skin allograft rejection. The model involved transplanting skin allografts from C57BL/6 mice onto the wound bed of Balb/c mice. The results demonstrated a significant dose-dependent prolongation of skin allograft survival in animals treated with the ethyl acetate extract. FACS analysis revealed that treatment with the extract (200 mg/kg) led to an immature state of dendritic cells and stimulated the differentiation of CD4^+^CD25^+^ Tregs. Moreover, the extract efficiently reduced T-bet gene expression and spleen lymphocyte proliferation in treated mice. In comparison to the model control, recipients treated with the extract exhibited significant downregulation of Th1 cytokines (IL-2, IFN-*γ*) and a notable increase in Th2 cytokine (IL-10) levels in the serum, with a dose-related pattern. These findings suggest that the ethyl acetate extract has anti-allograft rejection properties by promoting CD4+CD25+ Tregs differentiation and maintaining the immaturity of dendritic cells, thereby inducing a stable immunological tolerance state. This highlights its potential for the treatment of autoimmune diseases [14].

### 7.3. Hypoglycemic and Hypolipidemic Activity

To investigate the effects of total coumarins on diabetes, eight-week-old non-obese diabetic (NOD) mice were divided into four groups: a control group and low-dose (37.5 mg/kg), middle-dose (75 mg/kg), and high-dose (150 mg/kg) total coumarin treatment groups. The results demonstrated that treatment with total coumarins for four weeks significantly inhibited insulitis, increased pancreatic islet number, delayed the onset, and reduced the development of diabetes by twenty-six weeks of age in NOD mice compared to untreated control mice. Total coumarins also suppressed spleen T-lymphocyte proliferation, induced a Th2-biased cytokine response, promoted the generation of CD4^+^CD25^+^Foxp3^+^ Tregs, and increased Foxp3 mRNA expression. Furthermore, dendritic cells treated with total coumarins exhibited low expression of MHC class II and CD86 molecules. The expressions of the TLR4 gene and protein expressions in the spleen, thymus, and pancreas were downregulated in the groups treated with total coumarins. Key molecules involved in the downstream signaling cascades of TLR4, such as myeloid differentiation factor 88 (MyD88), NF-*κ*B, IL-1*β*, TRIF, TRAM, IRF-3, and IFN-*β*, all showed significant decreases in the total coumarins groups. This suggests that total coumarins inhibit both MyD88-dependent and -independent pathways of TLR4. At the cellular level, TLR4 protein expression was found to be downregulated by total coumarins in dendritic cells but not in Tregs. Furthermore, total coumarins enhanced the role of dendritic cells, rather than Tregs, in negative immune regulation in vitro. This effect on dendritic cell immune function was reversed by anti-TLR4 antibody. Therefore, the total coumarins from *L. bulbifera* can prevent autoimmune diabetes in mice by inhibiting the TLR4 signaling pathway [53].

To establish a model of insulin resistance type II diabetes, BALB/c mice were fed a high-fat diet and injected with small doses of STZ. The effects of different concentrations of total flavonoids of *L. bulbifera* (25, 50, 100 mg/kg) on the blood glucose concentration of the diabetic model were observed through daily intragastric administration. The results indicated that the total flavonoids group significantly reduced blood sugar levels in mice compared to the model group. Pancreatic HE staining showed no significant difference between the groups. The low-dose group demonstrated a significant effect in reducing triglycerides, total cholesterol, and the insulin resistance index. It also improved glucose tolerance in insulin-resistant mice. Insulin measurement results showed a significant increase in insulin levels only in the high-dose group. SOD and MDA levels did not show significant changes in any of the groups. Additionally, immunoblotting results for insulin receptors and PPAR-*γ* showed that the low-dose group of total flavonoids increased the expression of insulin receptor levels. These results demonstrate that total flavonoids exert a hypoglycemic and hypolipidemic effect by upregulating insulin receptor levels and increasing insulin sensitivity rather than affecting the free radical pathway [54].

In another study, male Kunming mice were fed a high-fat diet for two weeks to establish a model of hypercholesterolemia. *L. bulbifera* was extracted and separated using macroporous resin to obtain four fractions: water fraction, 30% ethanol fraction, 70% ethanol fraction, and 95% ethanol fraction. Each fraction was administered by gavage at a dose of 40 mg/g, and serum biochemical indicators were measured after four weeks. Liver sections were stained for observation. The experimental results showed that both the 30% ethanol fraction and 70% ethanol fraction significantly reduced body weight and serum levels of total cholesterol, low-density lipoprotein cholesterol, and MDA in hypercholesterolemic mice. They also increased the levels of SOD in experimental hypercholesterolemic mice. Staining results of mouse liver cells revealed that the liver tissue sections of mice treated with the 30% ethanol fraction and 70% ethanol fraction showed normal liver cells around the central vein, indicating that these fractions could protect and repair the liver tissue of hypercholesterolemic mice. In summary, the 30% ethanol fraction and 70% ethanol fraction of *L. bulbifera* could regulate blood lipid metabolism in experimental hypercholesterolemic mice and significantly reduce their blood lipid levels [5].

### 7.4. Other Pharmacological Effects

The inhibitory effect of seventeen isolated compounds on human steroid 5*α*-reductase 2 (SRD5*α*2) was evaluated using molecular docking methods. The findings revealed that the compound with the most significant inhibition at the active sites of SRD5*α*2 was 5,7,3′-trihydroxy-4-methoxyisoflavone-7-*O*-*β*-*D*-glucopyranoside (**29**), followed by 5,7,4-trihydroxy-isoflavone-5-*O*-*β*-*D*-glucopyranoside (**25**), kaemferitrin (**43**), genistin (**26**), and apigenin (**3**). These results provide theoretical evidence supporting the application of *L. bulbifera* in the treatment of benign prostatic hyperplasia [11].

Thirteen flavonoids isolated from the aerial parts of *L. bulbifera* were evaluated for their inhibitory activity against N1 neuraminidase. Among them, kaempferol-3-*O*-*β*-*D*-glucopyranoside (**31**), kaemferitrin (**43**), and quercetin-3-*O*-*β*-*D*-6″-acetylglucopyranoside (**42**) (at concentrations of 50, 100, and 200 μmol/L) exhibited significantly stronger inhibitory effects compared to the other ten compounds. This suggests that the activity of flavonols surpasses that of flavonoids and isoflavones [2].

### 7.5. Toxicity

There are records indicating that *L. Bulbifera* has minor toxicity, although ethnic doctors generally consider it non-toxic [55]. Research reports have demonstrated that the oral administration of a water decoction and powder suspension of *L. bulbifera* to mice exhibited a minimum lethal dose greater than 50 g/kg and 1.67 g/kg, respectively [44]. In our previous oral acute toxicity experiments, we observed high safety when mice were administered *L. bulbifera* via gavage (2000 g/kg), as no mouse deaths occurred within 24 h [27].

## 8. Discussion

Firstly, this manuscript provides a comprehensive overview of the chemical composition of *L. bulbifera*, a traditional ethnomedicine. The analysis reveals that *L. bulbifera* is abundant in flavonoids and fatty acids, two crucial phytochemicals known for their potent antioxidant properties. These compounds exhibit the ability to neutralize free radicals, thereby mitigating cellular damage caused by oxidative stress [28,42]. Moreover, they also possess significant anti-inflammatory effects by effectively suppressing the release of inflammatory pathways and cytokines [29,56]. In fact, studies have found that flavonoids and phenolics could effectively ameliorate rheumatoid arthritis, a chronic inflammatory disorder [57]. Additionally, evidence suggests that fatty acids play a vital role in the prevention and treatment of rheumatoid arthritis [58]. Therefore, considering the aforementioned findings, it could be inferred that the therapeutic effects of *L. bulbifera* in mitigating rheumatic arthritis, fractures, and falling injuries are primarily attributed to its rich content of flavonoids and fatty acids.

Additionally, two important issues related to quality control need to be addressed. Firstly, there is variation in the methods used to determine the chemical components in *L. bulbifera*. Different compounds, such as *β*-sitosterol [43], flavonoids (isorhamnetin-3-*O*-*α*-*L*-rhamnopyranosyl-(1-2)-*β*-galactopyranoside (**47**), rutin (**48**) [26], and catechins [25]), and flavonoids in combination with phenylpropanoids [46], have been measured to assess the quality of *L. bulbifera*. However, these research studies lack systematicity, making it unclear which components truly reflect the quality of *L. bulbifera*. Secondly, the established indicators for quality control of *L. bulbifera* have not been based on their pharmacological substance basis and quality markers. As a result, the exclusive analysis of active ingredients is lacking, compromising the ability to accurately reflect and evaluate the quality of *L. bulbifera*. Given the increasing market demand for *L. bulbifera*, ensuring its safety and effectiveness from the source is crucial. To achieve this, researchers should explore the anti-inflammatory material basis of *L. bulbifera*, clarify its mechanism of action, and establish the relationship between its anti-inflammatory spectrum and effects. It is also important to screen and identify quality biomarkers that can faithfully represent the quality of *L. bulbifera*. Addressing these issues is vital in maintaining the stable and reliable quality of *L. bulbifera*, thus meeting the growing demand for this medicinal plant.

Furthermore, coumarins and flavonoids have been identified as significant components in the treatment of arthritis and inflammation, respectively [1,11,16]. These two phytochemicals exhibit distinct active properties, indicating that they play different roles in the treatment process. Therefore, we believe that the origin and specific medicinal parts of *L. bulbifera* represent the primary influencing factors. It is well-known that numerous environmental elements, including growth conditions, geographical location, and habitat, can result in variations in plant composition. Factors like plant growth environment, soil quality, climate conditions, and light intensity may vary across different regions, leading to diverse chemical compositions and contents in the same plant species. Consequently, medicinal plants grown in different habitats may exhibit dissimilar ingredient profiles and quantities, potentially resulting in varied pharmacological and clinical effects within different regions. Additionally, the medicinal parts utilized can significantly impact the therapeutic outcomes. Our previous investigations, supported by the literature, have demonstrated that the roots possess superior antioxidant capacity compared to the aerial parts [27]. However, previous studies have employed a variety of medicinal parts, including roots [1], aerial parts [11], and the whole herb [25], contributing to disparate findings.

Moving forward, several crucial avenues of research should be pursued regarding *L. bulbifera*. Firstly, a more extensive exploration of its chemical composition is warranted to elucidate the specific substances responsible for its pharmacological effects. Secondly, a comprehensive analysis of its pharmacological mechanisms should be conducted to offer theoretical guidance and technical support for drug development and clinical application. Subsequently, quality control measures must be implemented to ensure the consistency and reliability of therapeutic effects. Finally, it is essential to systematically validate and optimize traditional applications of *L. bulbifera*, harnessing its full potential and broadening its prospects for practical use.

## 9. Conclusions

However, there is currently a lack of comprehensive and detailed documentation on the ethnomedicinal uses, geographical distribution, botanical description, phytochemistry, pharmacology, and quality control of *L. bulbifera*. Consequently, the primary objective of this review is to comprehensively explore existing research on *L. bulbifera* by examining multiple databases and addressing these aforementioned aspects. Furthermore, this review will identify potential areas for future research, such as isolating and identifying additional compounds found in *L. bulbifera*, conducting more extensive pharmacological evaluations, elucidating its mechanisms of action, and ultimately establishing a more robust quality control system. The outcomes of this research will serve as a solid basis for the quality control, product development, and clinical application of *L. bulbifera*.

## Figures and Tables

**Table 1:** Flavonoids isolated from *Laportea bulbifera*.

No.	Name	Source	Formula	Exact Theoretical M. W.	Characterization Method	Refs.
**1**	Daidzein	aerial parts, whole herb	C_15_H_10_O_4_	254.0579	^1^H NMR	[2]
					^1^H NMR	[24]
**2**	4′-Methoxyflavonol	roots	C_16_H_12_O_4_	268.0736	^1^H NMR, ^13^C NMR	[1]
**3**	Apigenin	aerial parts, roots	C_15_H_10_O_5_	270.0528	^1^H NMR, ^13^C NMR	[1]
					^1^H NMR, ^13^C NMR	[11]
**4**	5,6,7-Trihydroxyflavone	roots	C_15_H_10_O_5_	270.0528	HPLC-MS	[8]
**5**	Luteolin	roots, whole herb	C_15_H_10_O_6_	286.0477	^1^H NMR, ^13^C NMR, mp	[1]
					^13^C NMR, mp	[6]
**6**	Fisetin	roots	C_15_H_10_O_6_	286.0477	^1^H NMR, ^13^C NMR	[1]
**7**	Kaempferol	roots	C_15_H_10_O_6_	286.0477	^1^H NMR, ^13^C NMR	[1]
					HPLC-MS	[8]
**8**	Epicatechin	roots	C_15_H_14_O_6_	290.0790	^1^H NMR, ^13^C NMR	[1]
**9**	Catechin	roots	C_15_H_14_O_6_	290.0790	^1^H NMR, ^13^C NMR	[1]
**10**	5-Hydroxy-7,4′-dimethoxyflavone	roots	C_17_H_16_O_5_	300.0998	^1^H NMR, ^13^C NMR	[1]
**11**	Quercetin	roots, whole herb	C_15_H_10_O_7_	302.0427	^1^H NMR, ^13^C NMR	[1]
					UHPLC-ESI-Q-TOF-MS	[7]
**12**	(−)-Gallocatechin	roots	C_15_H_14_O_7_	306.0740	^1^H NMR, ^13^C NMR	[1]
**13**	Epigallocatechin	roots, whole herb	C_15_H_14_O_7_	306.0740	^1^H NMR, ^13^C NMR	[1]
					UV, mp, ESI-MS	[25]
**14**	(+)-4′,5,7-Trimethoxydihydroflavonol	roots	C_18_H_18_O_6_	330.1103	^1^H NMR, ^13^C NMR, ESI-MS	[1]
**15**	Naringenin trimethyl ether	roots	C_18_H_18_O_5_	314.1154	^1^H NMR, ^13^C NMR	[1]
**16**	Isorhamnetin	roots	C_16_H_12_O_7_	316.0583	^1^H NMR, ^13^C NMR	[1]
**17**	(+)-Dihydromyricetin	roots	C_15_H_12_O_8_	320.0532	^1^H NMR, ^13^C NMR	[1]
**18**	Tangeretin	roots	C_20_H_20_O_7_	372.1209	^1^H NMR, ^13^C NMR	[8]
**19**	Nobiletin	roots	C_21_H_22_O_8_	402.1315	^1^H NMR, ^13^C NMR	[1]
**20**	3,5,6,7,8,3′,4′-Heptamethoxyflavone	roots	C_22_H_24_O_9_	432.1420	^1^H NMR, ^13^C NMR	[8]
**21**	(−)-Epicatechin-3-*O*-gallate	roots	C_22_H_18_O_10_	442.0900	^1^H NMR, ^13^C NMR	[1]
**22**	(−)-Epigallocatechin 3-*O*-gallate	roots	C_22_H_18_O_11_	458.0849	^1^H NMR, ^13^C NMR	[1]
**23**	(−)-Gallocatechin 3-*O*-gallate	roots	C_22_H_18_O_11_	458.0849	^1^H NMR, ^13^C NMR	[1]
**24**	Daidzin	roots, aerial parts, whole herb	C_21_H_20_O_9_	416.1107	^1^H NMR, ^13^C NMR	[1]
					^1^H NMR	[2]
					^1^H NMR	[24]
**25**	5,7,4-Trihydroxy-isoflavone-5-*O*-*β*-*D*-glucopyranoside	aerial parts	C_21_H_20_O_10_	432.1056	^1^H NMR	[11]
**26**	Genistin	aerial parts	C_21_H_20_O_10_	432.1056	^1^H NMR	[11]
**27**	Kaempferol-7-*O*-*α*-*L*-rhamnoside	whole herb	C_21_H_20_O_10_	432.1056	mp, HR-MS, ^13^C NMR	[6]
**28**	Apigenin-7-*O*-*β*-*D*-glucopyranoside	aerial parts, whole herb	C_21_H_20_O_10_	432.1056	^1^H NMR, ^13^C NMR	[2]
					^1^H NMR, ^13^C NMR	[24]
**29**	5,7,3′-Trihydroxy-4-methoxyisoflavone-7-*O*-*β*-lucopyranoside	aerial parts	C_22_H_22_O_10_	446.1213	^1^H NMR	[11]
**30**	Luteoloside	roots	C_21_H_20_O_11_	448.1006	^1^H NMR, ^13^C NMR	[1]
**31**	Kaempferol-3-*O*-*β*-*D*-glucopyranoside	aerial parts, whole herb	C_21_H_20_O_11_	448.1006	^1^H NMR	[2]
					^1^H NMR	[24]
**32**	Luteolin-7-*O*-*β*-*D*-glucopyranoside	aerial parts, whole herb	C_21_H_20_O_11_	448.1006	^1^H NMR, ^13^C NMR	[2]
					^1^H NMR, ^13^C NMR	[24]
**33**	Quercetin-3-*O*-rhamnoside	roots	C_21_H_20_O_11_	448.1006	^1^H NMR, ^13^C NMR	[1]
**34**	Astragalin	roots	C_21_H_20_O_11_	448.1006	^1^H NMR, ^13^C NMR	[1]
**35**	Luteolin-7-galactoside	roots	C_21_H_20_O_11_	448.1006	UHPLC-MS	[8]
**36**	Pratensein-7-*O*-*β*-*D*-glucopyranoside	aerial parts, whole herb	C_22_H_22_O_11_	462.1162	^1^H NMR	[2]
					^1^H NMR	[24]
**37**	Isoquercitrin	whole herb, roots	C_21_H_20_O_12_	464.0955	UHPLC-ESI-Q-TOF-MS	[7]
**38**	Myricetin-3-*O*-*α*-*L*-rhamnopyranoside	roots	C_21_H_20_O_12_	464.0955	^1^H NMR, ^13^C NMR	[1]
**39**	Quercetin-3-alloside	roots	C_21_H_20_O_12_	464.0955	HPLC-MS	[8]
**40**	Hyperoside	roots, aerial parts, whole herb	C_21_H_20_O_12_	464.0955	^1^H NMR, ^13^C NMR	[1]
					^1^H NMR, ^13^C NMR	[2]
					^1^H NMR, ^13^C NMR	[24]
**41**	Quercetin-3-*O*-*β*-*D*-glucopyranoside	whole herb	C_21_H_20_O_12_	464.0955	^1^H NMR, ^13^C NMR, HR-MS	[24]
**42**	Quercetin-3-*O*-*β*-*D*-6″-acetylglucopyranoside	aerial parts	C_23_H_22_O_13_	506.1060	^1^H NMR	[2]
**43**	Kaemferitrin	aerial parts, whole herb	C_27_H_30_O_14_	578.1636	^1^H NMR	[2]
					mp, HR-MS, ^13^C NMR	[6]
					^1^H NMR, ^13^C NMR	[11]
**44**	Acaetin-7-*O*-rutinoside	aerial parts, whole herb	C_28_H_32_O_14_	592.1792	^1^H NMR, ^13^C NMR	[2]
					^1^H NMR, ^13^C NMR	[24]
**45**	Nicotiflorin	whole herb, roots	C_27_H_30_O_15_	594.1585	UHPLC-ESI-Q-TOF-MS	[7]
**46**	Isorhamnetin-3,7-*O*-*α*-*L*-dirhamnoside	roots	C_28_H_32_O_15_	608.1741	HPLC-MS	[8]
**47**	Isorhamnetin-3-*O*-*α*-*L*-rhamnopyranosyl-(1-2)-*β*-galactopyranoside	whole herb	C_28_H_32_O_16_	624.1690	^1^H NMR, ^13^C NMR	[10]
**48**	Rutin	roots, whole herb	C_27_H_30_O_16_	610.1534	^1^H NMR, ^13^C NMR	[1]
					UHPLC-ESI-Q-TOF-MS	[7]
**49**	Isorhamnetin-3-*O*-*α*-rhamnosyl-(1-2)-rhamnoside	whole herb	C_28_H_32_O_15_	608.1741	^1^H NMR, ^13^C NMR	[10]
**50**	Isorhamnetin-7-*O*-*α*-*L*-rhamnoside	roots	C_22_H_22_O_11_	462.1162	UHPLC-ESI-Q-TOF-MS	[27]
**51**	Isorhamnetin-3-*O*-*α*-*L*-rhamnoside	roots	C_22_H_22_O_11_	462.1162	UHPLC-ESI-Q-TOF-MS	[27]

UV: Ultraviolet spectrophotometry; ^13^C NMR: Carbon-13 nuclear magnetic resonance spectrometry; ^1^H NMR: Hydrogen-1 nuclear magnetic resonance spectrometry; HPLC-MS: High-performance liquid chromatography–mass spectrometry; HR-MS: High-resolution mass spectrometry; mp: Melting point; ESI-MS: Electrospray ionization mass spectrometry; UHPLC-ESI-Q-TOF-MS: Ultra high performance liquid chromatography–electrospray ionization–quadrupole–time of flight–mass spectrometry.

**Table 2:** Phenolics isolated from *Laportea bulbifera*.

No.	Name	Source	Formula	Exact Theoretical M. W.	Characterization Method	Refs.
**52**	Phloroglucinol	whole herb	C_6_H_6_O_3_	126.0317	mp, UV, ESI-MS, ^1^H NMR, ^13^C NMR	[25]
**53**	*p*-Hydroxybenzoic acid	roots, aerial parts	C_7_H_6_O_3_	138.0317	^1^H NMR, ^13^C NMR	[1]
					^1^H NMR, ^13^C NMR	[11]
**54**	Vanillic acid	roots, whole herb	C_8_H_8_O_4_	168.0423	^1^H NMR, ^13^C NMR	[1]
					^1^H NMR, ^13^C NMR	[4]
**55**	2-Hydroxy-3-(*O*-hydroxyphenyl) propanoic acid	roots	C_9_H_10_O_4_	182.0579	^1^H NMR, ^13^C NMR	[1]
**56**	Ethyl 3,4-dihydroxybenzoate	whole herb	C_9_H_10_O_4_	182.0579	mp, ^1^H NMR, ^13^C NMR	[6]
**57**	Ethyl gallate	whole herb	C_9_H_10_O_5_	198.0528	mp, HR-MS, ^1^H NMR, ^13^C NMR	[6]
**58**	C-Veratroylglycol	roots	C_10_H_12_O_5_	212.0685	^1^H NMR, ^13^C NMR	[8]
**59**	Flavokawain A	roots	C_18_H_18_O_5_	314.1154	^1^H NMR, ^13^C NMR	[1]
**60**	(+)-5,5-Dimethyl-5,6a,7,12a-tetrahydroisochromeno[4,3-b]chromene-2,3,4,8,10-pentaol	roots	C_18_H_18_O_7_	346.1053	^1^H NMR, ^13^C NMR, ESI-MS, ${\left[\alpha \right]}_{D}^{20}$	[1]
**61**	(−)-5,5-Dimethyl-5,6a,7,12a-tetrahydroisochromeno[4,3-b]chromene-2,3,4,8,10-pentaol	roots	C_18_H_18_O_7_	346.1053	^1^H NMR, ^13^C NMR, ESI-MS, ${\left[\alpha \right]}_{D}^{20}$	[1]
**62**	(+)-Vibruresinol	roots	C_20_H_24_O_6_	360.1573	^1^H NMR, ^13^C NMR	[1]
**63**	Piceid	roots	C_20_H_22_O_8_	390.1315	^1^H NMR, ^13^C NMR	[1]
**64**	Phloridzin	roots	C_21_H_24_O_10_	436.1369	^1^H NMR, ^13^C NMR	[1]
**65**	(+)-Isolariciresinol 9′-*O*-glucoside	whole herb	C_26_H_34_O_11_	522.2101	mp, HR-MS, ^13^C NMR	[6]
**66**	Salicylic acid	roots	C_7_H_6_O_3_	138.0317	UHPLC-ESI-Q-TOF-MS	[27]
**67**	Schizandriside	roots	C_25_H_32_O_10_	492.1995	UHPLC-ESI-Q-TOF-MS	[27]

UV: Ultraviolet spectrophotometry; UHPLC-ESI-Q-TOF-MS: Ultra high performance liquid chromatography–electrospray ionization–quadrupole–time of flight–mass spectrometry; mp: Melting point; HR-MS: High-resolution mass spectrometry; IR: Infrared spectroscopy; ^13^C NMR: Carbon-13 nuclear magnetic resonance spectrometry; ^1^H NMR: Hydrogen-1 nuclear magnetic resonance spectrometry; ESI-MS: Electrospray ionization mass spectrometry.

**Table 3:** Nitrogen compounds isolated from *Laportea bulbifera*.

No.	Name	Source	Formula	Exact Theoretical M. W.	Characterization Method	Refs.
**68**	Uracil	aerial parts	C_4_H_4_N_2_O_2_	112.0273	^1^H NMR	[11]
**69**	6-Hydroxypurine	aerial parts	C_5_H_4_N_4_O	136.0385	^1^H NMR	[11]
**70**	Quinolin-2(1H)-one	whole herb	C_9_H_7_NO	145.0528	UHPLC-Q-TOF-MS/MS	[5]
**71**	1H-Indole-3-carboxylic acid	aerial parts	C_9_H_7_NO_2_	161.0477	^1^H NMR	[11]
**72**	6-Hydroxy-5-methoxy-1H-indole-2-carboxylic acid	roots	C_10_H_9_NO_4_	207.0532	HPLC-MS	[8]
**73**	9-Ribofuranosyladenine	aerial parts	C_10_H_13_N_5_O_4_	267.0968	^1^H NMR	[11]
**74**	N2-Fructopyranosylarginine	roots	C_12_H_24_N_4_O_7_	336.1645	UHPLC-ESI-Q-TOF-MS	[27]
**75**	Choline	roots	C_5_H_14_NO^+^	104.1070	UHPLC-ESI-Q-TOF-MS	[27]

^1^H NMR: Hydrogen-1 nuclear magnetic resonance spectrometry; UHPLC-Q-TOF-MS/MS: Ultra high performance liquid chromatography–quadrupole–time of flight–mass spectrometry/mass spectrometry; HPLC-MS: High-performance liquid chromatography–mass spectrometry; UHPLC-ESI-Q-TOF-MS: Ultra high performance liquid chromatography–electrospray ionization–quadrupole–time of flight–mass spectrometry.

**Table 4:** Steroids isolated from *Laportea bulbifera*.

No.	Name	Source	Formula	Exact Theoretical M. W.	Characterization Method	Refs.
**76**	Ergosta-4,6,8(14),22-tetraen-3-one	whole herb	C_28_H_40_O	392.3079	EI-MS, ^1^H NMR, ^13^C NMR	[10]
**77**	Sitostenone	whole herb	C_29_H_48_O	412.3705	EI-MS, ^1^H NMR, ^13^C NMR	[10]
**78**	(+)-Cabralealactone	roots	C_27_H_42_O_3_	414.3134	^1^H NMR, ^13^C NMR	[1]
**79**	*β*-Sitosterol	aerial parts, roots	C_29_H_50_O	414.3862	^1^H NMR, mp, EI-MS	[9]
					^1^H NMR, ^13^C NMR	[11]
**80**	Stigmasta-4,22-diene-3,6-dione	whole herb	C_29_H_44_O_2_	424.3341	EI-MS, ^1^H NMR, ^13^C NMR	[10]
**81**	Stigmast-4-ene-3,6-dione	whole herb	C_29_H_46_O_2_	426.3498	EI-MS, ^1^H NMR, ^13^C NMR	[10]
**82**	7-Keto-*β*-Sitosterol	roots	C_29_H_48_O_2_	428.3654	^1^H NMR, ^13^C NMR	[1]
**83**	Sumaresinolic acid	roots	C_30_H_48_O_4_	472.3553	HPLC-MS	[8]
**84**	Asiatic acid	roots	C_30_H_48_O_5_	488.3502	HPLC-MS	[8]
**85**	*β*-Daucosterol	aerial parts, roots, whole herb	C_35_H_60_O_6_	576.4390	^1^H NMR, mp, EI-MS	[9]
					^1^H NMR, ^13^C NMR	[11]

^13^C NMR: Carbon-13 nuclear magnetic resonance spectrometry; ^1^H NMR: Hydrogen-1 nuclear magnetic resonance spectrometry; mp: Melting point; EI-MS: Electron impact mass spectrometry; HPLC-MS: High-performance liquid chromatography–mass spectrometry.

**Table 5:** Terpenoids isolated from *Laportea bulbifera*.

No.	Name	Source	Formula	Exact Theoretical M. W.	Characterization Method	Refs.
**86**	*α*-Ionol	aerial parts	C_13_H_20_O_3_	224.1412	^1^H NMR, ^13^C NMR	[11]
**87**	Genipin	roots	C_11_H_14_O_5_	226.0841	HPLC-MS	[8]
**88**	Nigranoic acid	roots	C_30_H_46_O_4_	470.3396	HPLC-MS	[8]

^13^C NMR: Carbon-13 nuclear magnetic resonance spectrometry; ^1^H NMR: Hydrogen-1 nuclear magnetic resonance spectrometry; HPLC-MS: High-performance liquid chromatography–mass spectrometry.

**Table 6:** Coumarins isolated from *Laportea bulbifera*.

No.	Name	Source	Formula	Exact Theoretical M. W.	Characterization Method	Refs.
**89**	Coumarin	whole herb	C_9_H_6_O_2_	146.0368	UHPLC-Q-TOF-MS/MS	[5]
**90**	7-Methoxy-2H-chromen-2-one	whole herb, roots	C_10_H_8_O_3_	176.0473	^1^H NMR, ^13^C NMR	[1]
					UHPLC-QTOF-MS/MS	[5]
**91**	3,6-Dihydroxycoumarin	whole herb	C_9_H_6_O_4_	178.0266	UHPLC-Q-TOF-MS/MS	[5]
**92**	4-Hydroxy-6-methoxy-2H-chromen-2-one	whole herb	C_10_H_8_O_4_	192.0423	UHPLC-Q-TOF-MS/MS	[5]
**93**	Scopoletin	aerial parts, whole herb	C_10_H_8_O_4_	192.0423	mp, ^1^H NMR, ^13^C NMR	[6]
					^1^H NMR	[11]
**94**	Scoparone	roots	C_11_H_10_O_4_	206.0579	^1^H NMR, ^13^C NMR	[1]
					^1^H NMR, ^13^C NMR	[4]
**95**	Dumetorine	whole herb	C_13_H_21_NO_2_	223.1572	UHPLC-Q-TOF-MS/MS	[5]
**96**	Isomeranzin	whole herb	C_15_H_16_O_4_	260.1049	^1^H NMR, ^13^C NMR	[30]
**97**	7,7′-Dimethoxy-6,6′-*bis*coumarin	roots	C_20_H_14_O_6_	350.0790	^1^H NMR, ^13^C NMR, IR, mp, HR-ESI-MS, UV	[4]
**98**	7,7′-Dihydroxy-6,6′ -dimethoxy-8,8′-*bis*coumarin	roots	C_20_H_14_O_8_	382.0689	^1^H NMR, ^13^C NMR, IR, mp, HR-ESI-MS, UV	[4]
**99**	6,6′,7,7′-Tetramethoxyl-8,8′-*bis*coumarin	roots	C_22_H_18_O_8_	410.1002	^1^H NMR, ^13^C NMR, IR, mp, HR-ESI-MS, UV	[4]

IR: Infrared spectroscopy; UV: Ultraviolet spectrophotometry; HR-ESI-MS: High-resolution electrospray ionization–mass spectrometry; mp: Melting point; UHPLC-Q-TOF-MS/MS: Ultra high performance liquid chromatography–quadrupole–time of flight–mass spectrometry/mass spectrometry; ^13^C NMR: Carbon-13 nuclear magnetic resonance spectrometry; ^1^H NMR: Hydrogen-1 nuclear magnetic resonance spectrometry.

**Table 7:** Phenylpropanoids isolated from *Laportea bulbifera*.

No.	Name	Source	Formula	Exact Theoretical M. W.	Characterization Method	Refs.
**100**	*trans*-Cinnamic acid	roots	C_9_H_8_O_3_	164.0473	HPLC-MS	[8]
**101**	*Z*-*p*-Hydroxy-cinnamic acid	roots	C_9_H_8_O_3_	164.0473	^1^H NMR, ^13^C NMR	[8]
**102**	*trans*-*p*-Hydroxycinnamic acid	aerial parts, whole herb	C_9_H_8_O_3_	164.0473	mp, HR-MS, ^13^C NMR	[6]
					^1^H NMR	[11]
**103**	*cis*-Hydroxycinnamic acid	aerial parts	C_9_H_8_O_3_	164.0473	^1^H NMR	[11]
**104**	Methyl-*trans*-4-hydroxycinnamate	aerial parts	C_10_H_10_O_3_	178.0630	^1^H NMR	[11]
**105**	4-Hydroxy-3-methoxycinnamaldehyde	roots	C_10_H_10_O_3_	178.0630	HPLC-MS	[8]
**106**	Caffeic acid	roots	C_9_H_8_O_4_	180.0423	^1^H NMR, ^13^C NMR	[30]
**107**	Ferulic acid	roots	C_10_H_10_O_4_	194.0579	HPLC-MS	[8]
**108**	Danshensu	roots	C_9_H_10_O_5_	198.0528	HPLC-MS	[8]
**109**	Sinapic acid	roots	C_11_H_12_O_5_	224.0685	HPLC-MS	[8]
**110**	Neochlorogenic acid	whole herb, roots	C_16_H_18_O_9_	354.0951	UHPLC-ESI-Q-TOF-MS	[7]
					LC-MS/MS	[26]
**111**	Chlorogenic acid	whole herb, roots	C_16_H_18_O_9_	354.0951	UHPLC-ESI-Q-TOF-MS	[7]
					LC-MS/MS	[26]
**112**	4-*O*-caffeoylquinic acid	whole herb, roots	C_16_H_18_O_9_	354.0951	UHPLC-ESI-Q-TOF-MS	[7]
					LC-MS/MS	[26]
**113**	Caffeic acid docosanoyl ester	roots	C_31_H_50_O_5_	502.3658	^1^H NMR, ^13^C NMR	[8]
**114**	Caffeic acid cinnamyl ester	roots	C_18_H_16_O_4_	296.1049	UHPLC-ESI-Q-TOF-MS	[27]
**115**	Secoisolariciresinol 9-*O*-*β*-*D*-glucopyranoside	roots	C_26_H_36_O_11_	524.2258	UHPLC-ESI-Q-TOF-MS	[27]
**116**	(*E*)-4-Coumaric acid	roots	C_9_H_8_O_3_	164.0473	UHPLC-ESI-Q-TOF-MS	[27]

^13^C NMR: Carbon-13 nuclear magnetic resonance spectrometry; ^1^H NMR: Hydrogen-1 nuclear magnetic resonance spectrometry; mp: Melting point; HR-MS: High-resolution mass spectrometry; HPLC-MS: High-performance liquid chromatography–mass spectrometry; UHPLC-ESI-Q-TOF-MS: Ultra high performance liquid chromatography–electrospray ionization–quadrupole–time of flight–mass spectrometry.

**Table 8:** Fatty acids and their derivatives isolated from *Laportea bulbifera*.

No.	Name	Source	Formula	Exact Theoretical M. W.	Characterization Method	Refs.
**117**	Hexadec-(4*Z*)-enoic acid	roots	C_16_H_30_O_2_	254.2246	^1^H NMR, ^13^C NMR	[8]
**118**	12-Hydroxypentanoic acid methyl ester	roots	C_15_H_30_O_3_	258.2195	^1^H NMR, ^13^C NMR	[8]
**119**	Methyl hexadec-9-enoate	whole herb	C_17_H_32_O_2_	268.2402	GC-MS	[25]
**120**	Methyl hexadecanoate	whole herb	C_17_H_34_O_2_	270.2559	GC-MS	[25]
**121**	Linoleic acid	roots	C_18_H_32_O_2_	280.2402	^1^H NMR, ^13^C NMR	[1]
**122**	Ethyl palmitate	whole herb	C_18_H_36_O_2_	284.2715	^1^H NMR, ^13^C NMR	[10]
**123**	Methyl linoleate	roots	C_19_H_34_O_2_	294.2559	^1^H NMR, ^13^C NMR	[1]
**124**	11-Octadecadienoic acid, methyl ester	whole herb	C_19_H_34_O_2_	294.2559	^1^H NMR, ^13^C NMR	[10]
**125**	Methyl oleate	whole herb	C_19_H_36_O_2_	296.2715	^1^H NMR, ^13^C NMR	[10]
**126**	Methyl stearate	whole herb	C_19_H_38_O_2_	298.2872	^1^H NMR, ^13^C NMR	[10]
**127**	Methyl (9*E*,11*E*)-8-oxooctadeca-9,11-dienoate	roots	C_19_H_32_O_3_	308.2351	^1^H NMR, ^13^C NMR	[1]
**128**	Ethyl linoleate	whole herb	C_20_H_36_O_2_	308.2715	^1^H NMR, ^13^C NMR	[10]
**129**	Ethyl Oleate	whole herb	C_20_H_38_O_2_	310.2872	GC-MS	[25]
**130**	(*Z*)-10-Eicosenoic acid	roots	C_20_H_38_O_2_	310.2872	^1^H NMR, ^13^C NMR	[8]
**131**	Methyl nonadecanoate	roots	C_20_H_40_O_2_	312.3028	^1^H NMR, ^13^C NMR	[1]
**132**	Nonanamide	roots	C_9_H_19_NO	157.1467	UHPLC-ESI-Q-TOF-MS	[27]
**133**	Linolenic acid	roots	C_18_H_30_O_2_	278.2246	UHPLC-ESI-Q-TOF-MS	[27]
**134**	Palmitic acid	roots	C_16_H_32_O_2_	256.2402	UHPLC-ESI-Q-TOF-MS	[27]
**135**	(*Z*)-9-Tetradecen-1-ol	roots	C_14_H_28_O	212.2140	UHPLC-ESI-Q-TOF-MS	[27]
**136**	1,18-Octadec-9-enedioic acid	roots	C_18_H_32_O_4_	312.2301	UHPLC-ESI-Q-TOF-MS	[27]
**137**	9(*Z*)-Octadecenamide	roots	C_18_H_35_NO	281.2719	^1^H NMR, ^13^C NMR	[8]
**138**	Octadecanedioic acid	roots	C_18_H_34_O_4_	314.2457	UHPLC-ESI-Q-TOF-MS	[27]
**139**	9-HpOTrE	roots	C_18_H_30_O_4_	310.2144	UHPLC-ESI-Q-TOF-MS	[27]
**140**	9-HOTrE	roots	C_18_H_30_O_3_	294.2195	UHPLC-ESI-Q-TOF-MS	[27]
**141**	Methyl nonadecanoate	roots	C_20_H_40_O_2_	312.3028	UHPLC-ESI-Q-TOF-MS	[27]
**142**	Fatty acid C18:5	whole herb	C_18_H_28_O_2_	276.2089	UHPLC-Q-TOF-MS/MS	[5]
**143**	Fatty acid C18:4	whole herb	C_18_H_30_O_2_	278.2246	UHPLC-Q-TOF-MS/MS	[5]
**144**	Fatty acid C18:8	whole herb	C_18_H_22_O_3_	286.1569	UHPLC-Q-TOF-MS/MS	[5]
**145**	Fatty acid C18:6	whole herb	C_18_H_26_O_3_	290.1882	UHPLC-Q-TOF-MS/MS	[5]
**146**	Fatty acid OH-C18:6	whole herb	C_18_H_26_O_3_	290.1882	UHPLC-Q-TOF-MS/MS	[5]
**147**	Atty acid OH-C18:5	whole herb	C_18_H_28_O_3_	292.2038	UHPLC-Q-TOF-MS/MS	[5]
**148**	Fatty acid C18:4	whole herb	C_18_H_30_O_3_	294.2195	UHPLC-Q-TOF-MS/MS	[5]
**149**	Fatty acid OH-C18:4	whole herb	C_18_H_30_O_3_	294.2195	UHPLC-Q-TOF-MS/MS	[5]
**150**	Fatty acid C18:4	whole herb	C_18_H_30_O_3_	294.2195	UHPLC-Q-TOF-MS/MS	[5]
**151**	Fatty acid C18:3	whole herb	C_18_H_32_O_3_	296.2351	UHPLC-Q-TOF-MS/MS	[5]
**152**	Fatty acid C20:3	whole herb	C_20_H_38_O_2_	310.2872	UHPLC-Q-TOF-MS/MS	[5]
**153**	Fatty acid C18:2	whole herb	C_18_H_34_O_5_	330.2406	UHPLC-Q-TOF-MS/MS	[5]
**154**	Fatty acid C22:6	whole herb	C_22_H_36_O_3_	348.2664	UHPLC-Q-TOF-MS/MS	[5]
**155**	Fatty acid OH-C22:5	whole herb	C_22_H_36_O_3_	348.2664	UHPLC-Q-TOF-MS/MS	[5]
**156**	Fatty acid 2OH-C20:2	whole herb	C_20_H_39_NO_4_	357.2879	UHPLC-Q-TOF-MS/MS	[5]
**157**	Amino fatty acid OH-C21:5	whole herb	C_21_H_35_NO_5_	381.2515	UHPLC-Q-TOF-MS/MS	[5]
**158**	Fatty acid OH-C30:9	whole herb	C_30_H_44_O_3_	452.3290	UHPLC-Q-TOF-MS/MS	[5]
**159**	Amino fatty acid 1	whole herb	C_18_H_37_NO_3_	315.2773	UHPLC-Q-TOF-MS/MS	[5]
**160**	Amino fatty acid 2	whole herb	C_18_H_39_NO_3_	317.2930	UHPLC-Q-TOF-MS/MS	[5]
**161**	Amino fatty acid 3	whole herb	C_19_H_37_NO_3_	327.2773	UHPLC-Q-TOF-MS/MS	[5]
**162**	Amino fatty acid 4	whole herb	C_20_H_43_NO_2_	329.3294	UHPLC-Q-TOF-MS/MS	[5]

GC-MS: Gas chromatography-mass spectrometry; UHPLC-Q-TOF-MS/MS: Ultra high performance liquid chromatography-quadrupole-time of flight-mass spectrometry/mass spectrometry; ^13^C NMR: Carbon-13 nuclear magnetic resonance spectrometry; ^1^H NMR: Hydrogen-1 nuclear magnetic resonance spectrometry.

**Table 9:** Others isolated from *Laportea bulbifera*.

No.	Name	Source	Formula	Exact Theoretical M. W.	Characterization Method	Refs.
**163**	Benzoic acid	roots	C_7_H_6_O_2_	122.0368	UHPLC-MS	[8]
**164**	5-Hydroxymethyl-2-furancarboxaldehyde	roots	C_6_H_6_O_3_	126.0317	^1^H NMR, ^13^C NMR	[8]
**165**	Malic acid	roots	C_4_H_6_O_5_	134.0215	HPLC-MS	[8]
**166**	Citric acid	roots	C_6_H_8_O_7_	192.0270	HPLC-MS	[8]
**167**	*Bis*(5-formylfurfuryl) ether	whole herb	C_12_H_10_O_5_	234.0528	^1^H NMR, ^13^C NMR	[30]
**168**	1′4-Diphenyl-1′4-butanedione	roots	C_16_H_14_O_2_	238.0994	^1^H NMR, ^13^C NMR, mp, EI-MS	[9]
**169**	1-(2-Phenylcarbonyloxyacetyl) benzene	roots	C_15_H_12_O_3_	240.0786	^1^H NMR, ^13^C NMR, mp, EI-MS	[9]
**170**	2,2′-Oxy-*bis*(1-phenylethanol)	roots	C_16_H_18_O_3_	258.1256	^1^H NMR, ^13^C NMR, mp, EI-MS	[9]
**171**	Dibutyl phthalate	whole herb	C_16_H_22_O_4_	278.1518	GC-MS	[25]
					^1^H NMR, ^13^C NMR	[30]
**172**	Phthalic acid, isobutyl nonyl ester	whole herb	C_21_H_32_O_4_	348.2301	GC-MS	[25]
**173**	Dioctyl phthalate	whole herb	C_24_H_38_O_4_	390.2770	^1^H NMR, ^13^C NMR	[30]
**174**	*Bis*(2-propylpentyl) phthalate	whole herb	C_24_H_38_O_4_	390.2770	GC-MS	[25]
**175**	Squalene	roots	C_30_H_50_	410.3913	^1^H NMR, ^13^C NMR	[1]
**176**	Betulaprenol 9	whole herb	C_45_H_74_O	630.5740	^1^H NMR, ^13^C NMR, EI-MS	[10]
**177**	Betulaprenol 8	whole herb	C_40_H_66_O	562.5114	^1^H NMR, ^13^C NMR, EI-MS	[10]
**178**	*L*-Proline	roots	C_5_H_9_NO_2_	115.0633	UHPLC-ESI-Q-TOF-MS	[27]
**179**	*L*-Tyrosine	roots	C_9_H_11_NO_3_	181.0739	UHPLC-ESI-Q-TOF-MS	[27]
**180**	Phenylalanine	roots	C_9_H_11_NO_2_	165.0790	UHPLC-ESI-Q-TOF-MS	[27]
**181**	Creoside IV	roots	C_17_H_32_O_10_	396.1995	UHPLC-ESI-Q-TOF-MS	[27]
**182**	1,4-*Bis*(benzoyloxy)butane	roots	C_18_H_18_O_4_	298.1205	UHPLC-ESI-Q-TOF-MS	[27]
**183**	4-(3-Hydroxy-1-butyl) -3,5,5-trimethyl-2-cyclohexenone	roots	C_13_H_22_O_2_	210.1620	UHPLC-ESI-Q-TOF-MS	[27]
**184**	Heptyl 6-*O*-*α*-*L* -arabinopyranosyl-*β*-*D*-glucopyranoside	roots	C_18_H_34_O_10_	410.2152	UHPLC-ESI-Q-TOF-MS	[27]
**185**	3×Leu-3H_2_O	whole herb	C_18_H_33_N_3_O_3_	339.2522	UHPLC-Q-TOF-MS/MS	[5]
**186**	Leu-Leu-Asp-Val-Leu-Met-Pro-Leu-Leu-9H_2_O	whole herb	C_49_H_85_N_9_O_11_S	1007.6089	UHPLC-Q-TOF-MS/MS	[5]
**187**	Leu-Leu-Asp-Val-Leu-Leu-Pro-Leu-Met-9H_2_O	whole herb	C_49_H_85_N_9_O_11_S	1007.6089	UHPLC-Q-TOF-MS/MS	[5]
**188**	Leu-Leu-Glu-Leu-Leu-Val-Pro-Met-Leu-9H_2_O	whole herb	C_50_H_87_N_9_O_11_S	1021.6246	UHPLC-Q-TOF-MS/MS	[5]
**189**	Leu-Leu-Val-Cit-Leu-Val-Asp-Leu-Met-9H_2_O	whole herb	C_49_H_87_N_11_O_12_S	1053.6256	UHPLC-Q-TOF-MS/MS	[5]

^13^C NMR: Carbon-13 nuclear magnetic resonance spectrometry; ^1^H NMR: Hydrogen-1 nuclear magnetic resonance spectrometry; UHPLC-Q-TOF-MS/MS: Ultra high performance liquid chromatography–quadrupole–time of flight–mass spectrometry/mass spectrometry; GC-MS: Gas chromatography–mass spectrometry; EI-MS: Electron impact mass spectrometry; UHPLC-MS: Ultra high performance liquid chromatography–quadrupole–mass spectrometry; HPLC-MS: High-performance liquid chromatography–mass spectrometry; UHPLC-ESI-Q-TOF-MS: Ultra high performance liquid chromatography–electrospray ionization–quadrupole–time of flight–mass spectrometry.

## Data Availability

Data are contained within the article.
